# Strict *De Novo* Methylation of the 35S Enhancer Sequence in Gentian

**DOI:** 10.1371/journal.pone.0009670

**Published:** 2010-03-23

**Authors:** Kei-ichiro Mishiba, Satoshi Yamasaki, Takashi Nakatsuka, Yoshiko Abe, Hiroyuki Daimon, Masayuki Oda, Masahiro Nishihara

**Affiliations:** 1 Graduate School of Life and Environmental Sciences, Osaka Prefecture University, Sakai, Osaka, Japan; 2 Iwate Biotechnology Research Center, Kitakami, Iwate, Japan; University of Leeds, United Kingdom

## Abstract

A novel transgene silencing phenomenon was found in the ornamental plant, gentian (*Gentiana triflora* × *G. scabra*), in which the introduced Cauliflower mosaic virus (CaMV) 35S promoter region was strictly methylated, irrespective of the transgene copy number and integrated loci. Transgenic tobacco having the same vector did not show the silencing behavior. Not only unmodified, but also modified 35S promoters containing a 35S enhancer sequence were found to be highly methylated in the single copy transgenic gentian lines. The 35S core promoter (−90)-introduced transgenic lines showed a small degree of methylation, implying that the 35S enhancer sequence was involved in the methylation machinery. The rigorous silencing phenomenon enabled us to analyze methylation in a number of the transgenic lines in parallel, which led to the discovery of a consensus target region for *de novo* methylation, which comprised an asymmetric cytosine (CpHpH; H is A, C or T) sequence. Consequently, distinct footprints of *de novo* methylation were detected in each (modified) 35S promoter sequence, and the enhancer region (−148 to −85) was identified as a crucial target for *de novo* methylation. Electrophoretic mobility shift assay (EMSA) showed that complexes formed in gentian nuclear extract with the −149 to −124 and −107 to −83 region probes were distinct from those of tobacco nuclear extracts, suggesting that the complexes might contribute to *de novo* methylation. Our results provide insights into the phenomenon of sequence- and species- specific gene silencing in higher plants.

## Introduction

Epigenetic regulation of gene expression has been uncovered by recent progress in genome research. In higher plants, many regulatory factors for epigenetic regulation have been isolated, mainly from Arabidopsis [1], [2]. Studies on transgene silencing in higher plants have contributed to the elucidation of epigenetic regulation [3]. In plants, transgenes can be silenced by both post-transcriptional gene silencing (PTGS) and transcriptional gene silencing (TGS), according to presence or absence of transcription [4]. Vaucheret and Fagard [4] classified TGS into *trans*-TGS, which is often induced by endogenous sequences in the genome similar to transgenes, and *cis*-TGS, which occurs in cases where the transgene is duplicated or is inserted into a transcriptionally inactive region subject to silencing. In both cases, hypermethylation of the transgene generally occurs in the transgene promoter region. That is, *de novo* methylation must have been triggered in such TGS silencing at some point after integration of the transgene into the host genome.

The mechanisms of sequence specific targeting of DNA methylation have been investigated by various researchers in recent years [1], [5], [6]. RNA-directed DNA methylation (RdDM), which is associated with the establishment of DNA methylation patterns and the initiation of gene silencing, has been discovered in plants [7]. Although there are potential alternative mechanisms to RdDM for introducing sequence specific DNA methylation, the causal factors for such phenomena are still unclear [8], [9].

Even if the fundamental mechanisms of epigenetic regulation are shared in higher plant species, it is likely that particular characteristics of the epigenetic machinery have diversified in some plant species during evolution. One way to detect the divergence of epigenetic regulation is a comparative study of transgene silencing in different plant species; however, no study has focused on such an aspect. This could be due to the difficulty in providing a number of transformants sufficient for appropriate comparisons in most non-model plants. Nevertheless, different modes of transgene silencing might affect the screening efficiency of transformants in plant species, which is assumed to cause difficulty in obtaining transformants. This assumption is supported by a study of transgenic Mexican limes, which showed frequent transgene silencing after non-selective screening, whereas no silencing was found after selective culture [10].

Our previous study, using the ornamental floral plant, gentian (*Gentiana triflora* × *G. scabra*), showed that strict transgene silencing through cytosine methylation was observed in single copy T-DNA introduced transgenic plants [11]. No evidence of transgene silencing in single copy transgenic tobacco plants having the same T-DNA construct was observed; therefore, the silencing phenomenon might occur in a species-specific manner. Furthermore, the Cauliflower mosaic virus (CaMV) 35S promoter (35Spro) [12], [13] seemed to be the target for silencing in gentian, because methylated cytosines were frequently detected in the 35Spro region, and that transgenic gentian introduced with the *rolC* promoter did not exhibit silencing [11], [14]. However, it is difficult to discuss causal factor(s) for the silencing phenomenon, because the previous study used T-DNA constructs containing two 35Spro sequences and an endogenous MADS-box gene, *GtMADS4*. Hence, the possibility that the tandem structure of the 35Spro and/or the use of an endogenous gene might be a potent inducer for gentian silencing still remains. To clarify this, the present study used a vector containing a sole 35Spro connected to a *sGFP* reporter gene within the T-DNA region, and obtained transgenic gentian plants having single copies of the 35S construct within their genome.

We also studied *de novo* methylation using asymmetric sequence contexts, in which cytosine methylation is frequently observed in higher plants [15], [16]. Maintenance DNA methyltransferase (e.g. MET1 in Arabidopsis) recognizes hemimethylated CpG sites and methylates the cytosine in the unmethylated DNA strand [17]; therefore, the methylation status is likely to be retained at the CpG dinucleotide context. In plants, cytosine methylation on other sequence contexts, such as CpWpG (W is A or T) and asymmetric cytosine sequences (designated as CpHpH; H is A, C or T), is also found. Domains rearranged methyltransferase (DRM) class and chromomethylase (CMT) class of DNA methyltransferases in Arabidopsis are involved in methylation of non-CpG sequences [16]. DRM class methyltransferases have homology to mammalian Dnmt3 *de novo* methyltransferase, whereas CMT class methyltransferases are unique to plants and maintain non-CpG (primarily CpWpG) methylation [2], [18]. Asymmetric (CpHpH) methylation can only be maintained by *de novo* methylation [19], supporting the idea that monitoring of asymmetric methylation might reveal a “hot spot” region of *de novo* methylation in the plant genome. Conversely, the state of symmetric methylation is difficult to determine when *de novo* methylation has occurred.

Herein, we describe the strict sequence-specific transgene silencing phenomenon that occurs in gentian. A defined region of the 35S enhancer was revealed as a possible target sequence for the strict silencing apparatus by analysis of asymmetric methylation in transgenic plants having a single copy of unmodified- and modified 35S promoters. Silencing did not occur in transgenic tobacco, therefore the silencing machinery is thought to be specifically diversified in gentian and might contribute to genomic homeostasis against parasitic sequences.

## Results

### Production of single copy transgenic gentian and tobacco plants

**Figure 1:**
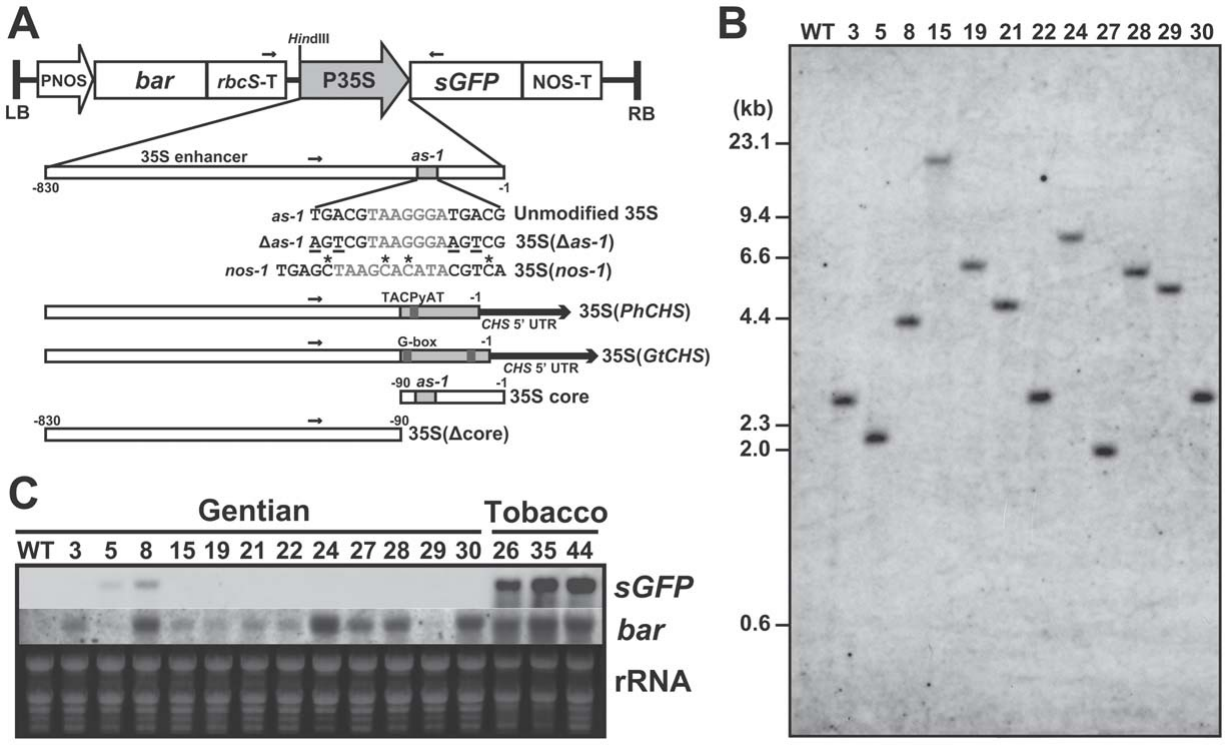
Structures of the T-DNA regions and molecular characteristics of transgenic gentians. (A) A T-DNA construct of the binary vector pSMABR35SsGFP (above) and schematic diagrams of the modified 35S promoters. Modified nucleotides are indicated as underlined letters [35S(Δ*as-1*)]. Altered cytosine residues are marked with asterisks [35S(*nos-1*)]. The gray regions indicate *CHS* promoter core regions of petunia [35S(*PhCHS*)] and gentian [35S(*GtCHS*)], respectively. 5′ UTRs of the *CHS* promoters are indicated as thick black arrows. Positions of primers used for bisulfite-PCR are indicated as small arrows. (B) Representation of single copy transgene in the unmodified 35S transgenic gentian plants by Southern blot analysis using *Hin*dIII-digested genomic DNAs with the *bar* gene probe. (C) Northern blots of *sGFP* and *bar* genes in leaf tissues of the single copy unmodified 35S transgenic gentian and tobacco plants. Wild-type (WT) gentian plant was also used as a control.

We produced transgenic gentian plants into which a single copy of the 35Spro was introduced by *Agrobacterium*-mediated transformation. The T-DNA region of the binary vector, pSMABR35SsGFP (Figure 1A), did not use any endogenous sequence from the gentian genome or any (inverted) repeat structure, unlike vectors used in our previous study [11]. Consequently, 21 independent transformant lines were obtained, of which 12 lines represented single copy lines, as confirmed by Southern blot analysis using *bar* and *sGFP* gene probes (Figure 1B, Table S1). For comparison, six lines of single copy transgenic tobacco (*Nicotiana tabacum* cv. SR1) plants transformed with the same construct were also screened from 31 lines produced. Among the gentian lines, two lines (#5 and #8) exhibited low expression of *sGFP* mRNA, but all other lines exhibited no *sGFP* expression in the leaf tissues of *in vitro* cultured plants (Figure 1C). In the leaves of all six single copy transgenic tobacco plants, strong *sGFP* expression was observed. The mRNA expression of the *bar* gene, which was used as a selection marker, varied among the transformants (Figure 1C). GFP fluorescence was observed in the transformed calli in an early stage after transformation (Figure S1), thus silencing of the *sGFP* transgene did not occur at the callus stage.

### Methylation status of the 35S-*sGFP* region

**Figure 2:**
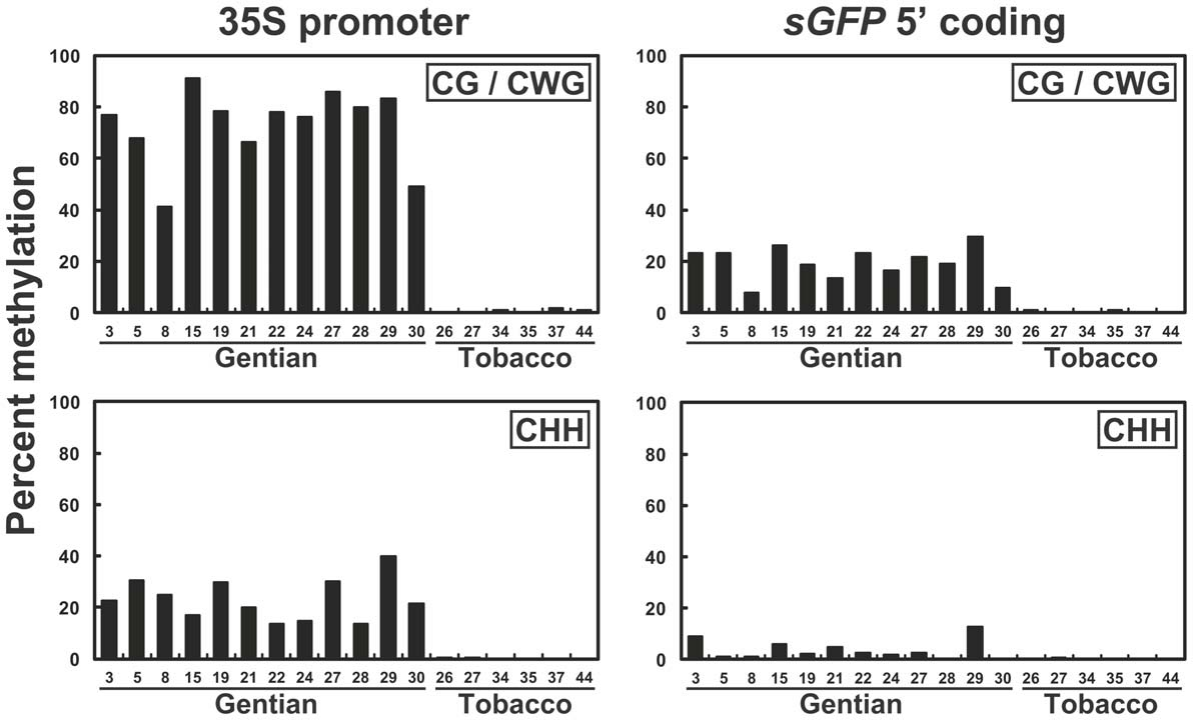
Methylation frequencies of the 35S and *sGFP* regions in the transgenic gentian and tobacco plants. Average percent methylation of CpG/CpWpG (upper panels) and CpHpH (lower panels) sites in the 35Spro (−257 to −1; left panels) and *sGFP* 5′ coding (+1 to +110; right panels) regions of the single copy unmodified 35S transgenic gentian and tobacco lines.

To investigate whether the 35Spro sequence underwent cytosine methylation in the transgenic plants, bisulfite genomic sequencing [20] was performed. To avoid incomplete bisulfite conversion in our genomic DNA samples, strict conditions (i.e. high-temperature for a long time, see Materials and Methods) were used, and methylation of the endogenous *CHS* promoter region [11] was evaluated for conversion efficiency (data not shown). Consequently, the bisulfite-treated products were found to be sufficiently converted (>99.8%). Accordingly, genomic DNAs derived from leaf tissues of all the single copy lines (12 gentians and six tobaccos) were subjected to bisulfite conversion (Table S1). The 35Spro with the *sGFP* coding region (from −257 to +110) was PCR-amplified (see Figure 1A), and at least eight independent clones were sequenced. Figure 2 shows the frequencies of methylated cytosines of each 35Spro and s*GFP* region analyzed from the transgenic gentian and tobacco lines. The cytosine methylation frequencies were divided into symmetric (CpG and CpWpG) and asymmetric (CpHpH) sequence contexts. All the gentian samples showed hypermethylation at CpG and CpWpG sites and moderate methylation frequencies at CpHpH sites in the 35Spro region, whereas no methylation was observed in the same region from the tobacco samples (Figure 2; see detail in Figures S2A and S2H). Compared with the 35S region, lower frequencies of CpG/CpWpG methylation of the *sGFP* region were observed in all gentian samples, and the methylation frequencies at CpG/CpWpG sites were correlated between the two regions in each gentian line (Figure 2). Within the promoter sequence, high methylation frequencies were commonly observed at a region 5′ to the *as-1* element (−82 to −62) [13], [21] (Figure S2A), which was consistent with our previous result using tandem 35S constructs [11].

### Modified 35S promoters introduced transgenic gentian

To confirm whether the *as-1* element is involved in *de novo* methylation in gentian, modified 35S promoters, which were substituted for the 35Spro of the pSMABR35SsGFP vector, were constructed (Figure 1A). The six different modified 35S constructs were introduced through *Agrobacterium*-mediated transformation and 194 lines of independent transformants were obtained. After copy-number estimation by Southern blot analysis, 14, 10, 11, 14, 10, and 15 lines for 35S(Δ*as-1*), 35S(*nos-1*), 35S(*PhCHS*), 35S(*GtCHS*), 35S core, and 35S(Δcore), respectively, were identified as single copy lines (Table S1). Subsequently, the methylation states of the modified promoter regions of all the single copy transgenic plant lines were determined using bisulfite analysis (Figure S2).

A point mutation of the *as-1* element (see Figure 1A) conferred a substantial reduction of GFP fluorescence in the 35S(Δ*as-1*) transformed gentian calli (Figure S1), supporting the hypothesis that *as-1* binding factor(s) might principally act for 35S transcription in gentian callus tissues at an early stage after transformation. On the other hand, replacement of the *nos-1* element [22] for the *as-1* element allowed high GFP expression in transformed calli (Figure S1), followed by suppression in the regenerated plant tissues (Figure S3A), which is comparable to the unmodified 35S lines. In either case, hypermethylation of the modified 35Spro regions was observed in all the 35S(Δ*as-1*) and 35S(*nos-1*) transgenic plant lines, as well as the unmodified 35S lines (Figures S2A–C).

As for the 35S(*PhCHS*) and 35S(*GtCHS*) lines, in which the 35S core region (−90) was replaced by the core regions of the *CHS* promoters from petunia [23] and gentian [24], respectively, strong GFP expression at the callus stage (Figure S1) and subsequent suppression in transgenic plant lines (Figures S3B and S3C) were also observed. Hypermethylation was also observed in the modified 35Spro regions in all transgenic plant lines analyzed (Figures S2D and S2E).

Surprisingly, all the 35S(Δcore) lines also exhibited hypermethylation of the introduced 35S enhancer region (−257 to −91), despite the fact that this construct lacks the 35S core promoter (−90) region (Figure S2F). On the contrary, hypomethylation of the 35S core region was observed in some 35S core lines (Figure S2G; see also Figure S3D for examples of *sGFP* and *bar* expressions in both lines). As a result, the 35S core region, including the *as-1* element, was considered to be dispensable for 35Spro-specific methylation in gentian.

### Analysis of *de novo* methylation using non-CpG/CpWpG cytosine sequences

**Figure 3:**
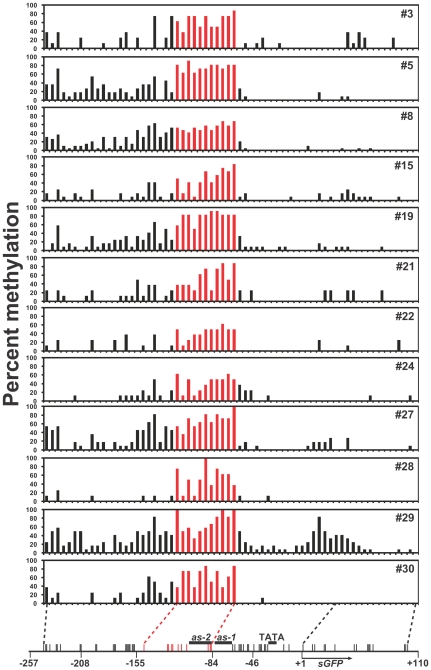
*De novo* methylation maps of the 35S-*sGFP* region in the single copy transgenic gentians. Percent methylation at CpHpH sites of 35S-*sGFP* region in the single copy unmodified 35S transgenic gentian lines (#3 to #30) is represented by bar charts. Positions of cytosines in CpHpH (vertical bars) motifs on the analyzed regions are represented below. Positions of cytosines from −148 to −85 are indicated by red bars.

To identify the possible target region of *de novo* methylation of the 35Spro, we focused on the asymmetric cytosine sequences from the bisulfite data obtained. Figure 3 shows a schematic representation of CpHpH methylation of the 35S-*sGFP* region in the unmodified 35S introduced gentian lines. Interestingly, distinct peaks, spanning 11 cytosines from positions −148 to −85 of the CpHpH methylation region, were shown in all the lines analyzed (Figure 3; indicated by red bars). High levels of CpHpH methylation were also observed within the region of the complementary strand (Figure S2I). In the remaining region, traces of methylated cytosines were scattered out the 5′ region of the analyzed sequence, with a slight peak around the cytosine at position −161. On the other hand, the 35S core region (comprising 17 cytosines from −66 to −12) and the adjoining *sGFP* 5′ region (15 cytosines from +1 to +101) rarely contained methylated cytosines except in #29 line that had a moderate peak in the region (Figure 3). From the NOS promoter to the *bar* 3′ region and the *rbcS* terminator to the 35S 5′ region, no or few CpHpH methylations (indicated by black bars) were observed in the unmodified 35S lines (#15 and #19), despite a certain level of CpG/CpWpG methylation (red and green bars, respectively) being detected in both lines (Figure S4).

**Figure 4:**
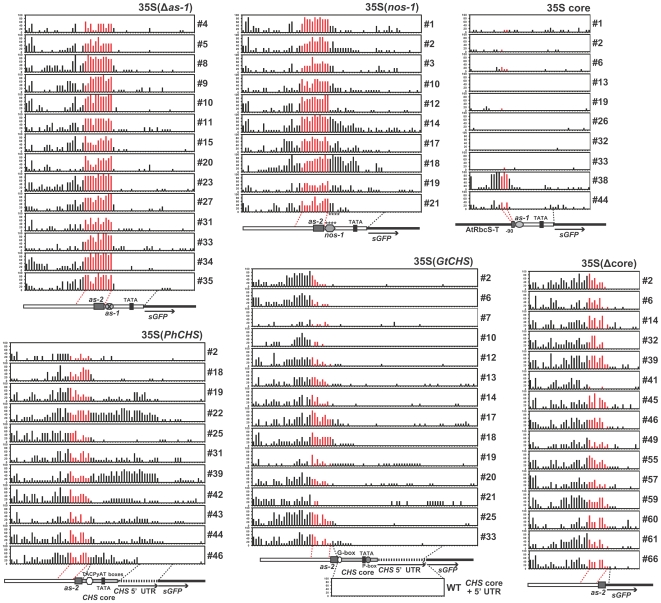
*De novo* methylation maps of the modified 35S promoter regions in the transgenic gentians. Percent methylation at CpHpH sites of the six different modified 35Spro regions in all of the single copy transgenic gentian lines tested are represented by bar charts. Analyzed regions of the modified 35S promoters (unmodified 35Spro sequences are indicated by white bars) with *sGFP* (black bar; indicated by arrows) are represented below each chart. Positions of *cis*-elements are indicated by marks. Regions of *CHS* core promoters and *CHS* 5′ UTRs are shown by gray and dotted bars, respectively. In the 35S(*GtCHS*) region, percent methylation of the corresponding endogenous *CHS* region from wild-type plants is also shown at the bottom. Positions of cytosines corresponding to the sequence from −148 to −85 of the unmodified 35Spro are indicated by red bars.

A distinct distribution of CpHpH methylation was also observed in the 35S(Δ*as-1*) transgenic lines. All 14 lines showed prominent CpHpH methylation peaks at the same regions (−148 to −85) as those in the unmodified 35S lines (Figure 4; indicated by red bars). In addition, most of the lines had moderate CpHpH methylation in the 35S 5′ region (−257 to −161) and slight methylation in the 35S core and *sGFP* regions, similar to the unmodified 35S lines.

### Altered *de novo* methylation patterns in the modified 35Spro

Despite the CpHpH methylation peak spanning −148 to −85 region being observed in all of 35S(*nos-1*) lines, their distributions of CpHpH methylation tended to extend towards the 35S core region, where the methylation levels were generally low in the unmodified 35S and 35S(Δ*as-1*) lines (Figure 4). The variability of the CpHpH methylation pattern was further characterized in the 11 35S(*PhCHS*) lines, in which two lines (#22 and #39) with strong CpHpH methylation patterns and six lines (#19, #31, #42, #43, #44, and #46) with moderate CpHpH methylation patterns were observed on the replaced petunia *CHS* core promoter region (Figure 4). On the other hand, no or very little CpHpH methylation was observed in the replaced gentian *CHS* core promoter region of all the 35S(*GtCHS*) lines. Among these *CHS*-core substituted lines, CpHpH methylation in the sequence corresponding to the −148 to −85 region [note three cytosines at the 3′ end of the region in the unmodified 35Spro were not contained in the 35S(*PhCHS*), 35S(*GtCHS*) and 35S(Δcore) promoters] of the unmodified 35Spro was also shown (Figure 4; indicated by red bars). However, the extent of methylation tended to be rather lower than those in the unmodified 35S, 35S(Δ*as-1*), and 35S(*nos-1*) lines.

Similar CpHpH methylation patterns to the *CHS*-core substituted lines were also observed in the 35S(Δcore) lines. All the lines had a moderate extent of CpHpH methylation in the 35S enhancer region. In the case of 10 lines of the 35S core lines, no (#13, #26, #32, #33) or very low amounts (#1, #2, #6, #19) of CpHpH methylation were predominantly observed, whereas moderate (#44) and strong (#38) CpHpH methylation was also detected (Figure 4).

### Increasing frequency of *de novo* methylation by culture of transgenic gentian plants

CpHpH methylation states of the 35S(Δcore) lines, which had been obtained after *Agrobacterium* inoculation on Jun. 7, 2005, were compared over a time-series of culture. Genomic DNAs from the *in vitro* cultured plants were obtained on Oct. 16, 2006 and Mar. 22, 2007 (11 and 16 months of culture after regeneration, respectively). An obvious increase in CpHpH methylation frequencies in the samples collected later (meanwhile, there was one time of subculturing the apical shoots) was found as compared with the corresponding lines collected earlier (Figure S5). The methylation frequencies of CpG/CpWpG sequences also increased during the five months of culture, synchronously with CpHpH methylation (data not shown).

### Identification of possible causal agents of the 35S *de novo* methylation

To identify possible causal agents for the *de novo* methylation of the 35S enhancer, we analyzed small RNA and genomic DNA in the wild-type and unmodified 35S transgenic gentian and tobacco plants. However, neither small RNA molecules (Figure S6A) nor genomic DNA sequences (Figure S6B) corresponding to the 35S enhancer could be detected in the gentian and tobacco plants.

### Gentian nuclear factors bind to the −149 to −124 region and the *as-2* element

**Figure 5:**
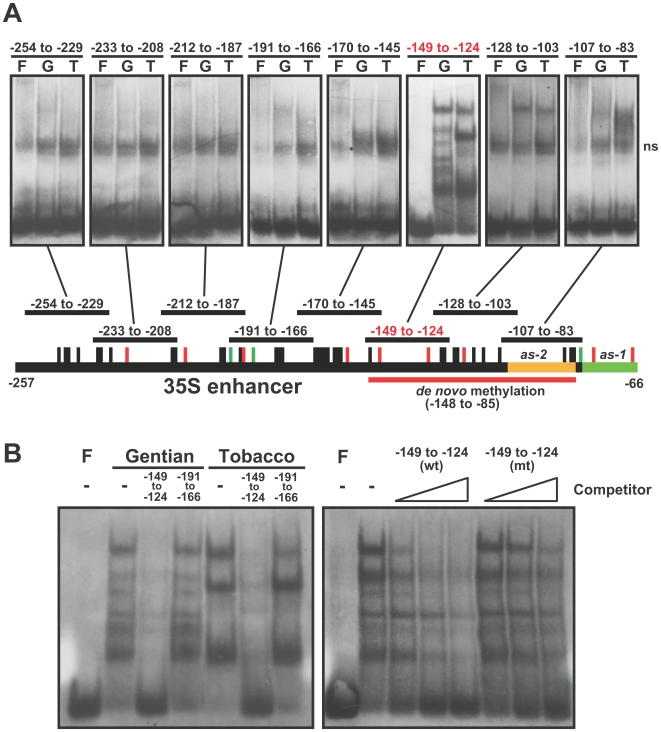
Gentian nuclear factors bounds to −149 to −124 region of the 35S enhancer. (A) Electrophoretic mobility shift assay (EMSA) using 26 bp probes (except −107 to −83 region with 25 bp) from each of eight different parts of the 35S enhancer region where bisulfite analysis was performed. Each panel shows three lanes (F, free probe; G, probe with gentian nuclear extract; T, probe with tobacco nuclear extract), and indicates each region at the top of the panel. ns, positions of non-specific signals. (B) Determination of binding affinity of gentian or tobacco nuclear extracts with the −149 to −124 region probe. The 100-fold molar excess of unlabeled wild-type −149 to −124 or −191 to −166 region competitors were incubated with the gentian or tobacco nuclear extracts (left panel). Binding reactions in the presence of 2-, 10-, and 30-fold molar excess of the wild-type (wt) or mutated (mt) −149 to −124 region competitors were performed in the presence or absence (F) of the gentian nuclear extract (right panel).

To explore other possible candidates, we tried to detect DNA binding factor(s) on the 35S enhancer region from the wild-type gentian nuclei extracts by electrophoretic mobility shift assay (EMSA). Using eight different 26-bp probes covering the −254 to −83 of the 35S enhancer region, distinctly different forms of complexes between gentian and tobacco nuclear extracts were identified using the −149 to −124 probe (Figure 5A). The probe was competed with unlabeled wild-type probe but not with the −191 to −166 or mutant (see Table S3) probes, indicating that the binding is sequence-specific (Figure 5B). In addition, a slightly retarded band was observed when the *as-2* [25] probe (−107 to −83) was used with gentian nuclear extract, whereas tobacco nuclear extract strongly bound to the probe.

## Discussion

### Species-specific 35S promoter silencing

The present study focused on single copy transgenic plant lines to avoid the copy-number effect [26], [27] and analyzed different genomic positions. We clearly showed that introduction of the single copy unmodified 35Spro was subject to silencing by hypermethylation in the transgenic gentian plants without exceptions. Although cytosine methylation was also observed in the NOS promoter, the frequency of methylation in the 35Spro region was demonstrably higher (Figure S4B). Consistent with these results, *bar* mRNA expression in some transgenic gentian lines (#8, #24, #27, #28 and #30) was comparable with that of tobacco, whereas *sGFP* mRNA expression of the transgenic gentians was considerably suppressed (Figure 1C). Reduced *bar* mRNA expression in other gentian lines was probably caused by symmetric methylation in the NOS promoter and/or *bar* gene coding region (Figure S4B), in which the methylation might occur by spreading from the 35S region [28]. In addition, transgenic gentian plants using promoters other than 35Spro showed stable transgene expression [11], [14], implying that the 35Spro sequence acts as a possible inducer of cytosine methylation in gentian. Suppression of *sGFP* mRNA transcription is thought to be mainly caused by the methylation of two cytosine residues in the *as-1* element, because a previous report also showed that the motif is sensitive to cytosine methylation in petunia [29]. Accordingly, the slight *sGFP* mRNA expression in the #8 and #5 lines might correlate with lower methylation frequencies (43.4 and 68.2%, respectively) at the two cytosines of the lines compared with those of the other lines (ranging from 68.8 to 100%).

Contrastingly, all the single copy transgenic tobacco lines showed strong s*GFP* expression with no methylation of the 35Spro sequence, which is consistent with the previous studies showing that 79 [30] and 10 [31] independent single copy Arabidopsis transgenic lines using 35Spro revealed high and stable expression, without exception. 35Spro is the most widely used promoter for driving transgenes, not only in dicots [32], but also in some monocots [33], [34], suggesting that the strict silencing phenomenon is specific to gentian. In some plant species, nevertheless, 35Spro failed to confer high expression [10], [35], [36]; further study would be needed to determine whether these species share a similar silencing machinery with gentian. To the best of our knowledge, the present study is the first attempt to determine the different epigenetic response to single copy transgenes in different plant species.

### Survey of asymmetric methylation to elucidate *de novo* methylation

The CpHpH methylation analysis allowed us to infer that the consensus target sequence for *de novo* methylation was the −148 to −85 region of the 35Spro. In the representation of methylation with all the cytosine sequence contexts (Figure S2A), high levels of methylation at the CpG/CpWpG sites were scattered, not only in the −148 to −85 region, but also in other regions including the *sGFP* coding region in some lines. Importantly, the presence of the symmetric methylation patterns was not stable among the transgenic lines, unlike the uniform pattern of the CpHpH methylation (Figure 3). Moderate and high levels of methylation at CpG/CpWpG sites were also observed in the *bar* coding and *rbcS* terminator to 35S 5′ regions, respectively, where distinct levels of CpHpH methylation were not observed (Figure S4). Thus, we hypothesized that the symmetric methylation of these regions was a consequence of methylation spreading from the *de novo* methylated −148 to −85 region.

Highly uniform *de novo* methylation of the 11 asymmetric cytosines spanning −148 to −85 were detected in all 26 unmodified 35S and 35S(Δ*as-1*) lines (Figures 3 and 4), suggesting that a strict sequence-specific *de novo* methylation occurred, regardless of the presence of the *as-1* element, the orientation of the DNA strands (Figure S2I), and genomic location. The frequencies of CpHpH methylation increased during culture (Figure S5); therefore, the establishment of stable *de novo* methylation status might require continuous growth and/or some developmental process. Although RdDM is known to be a major coordinator for sequence-specific *de novo* methylation in plants [37], alternative mechanisms probably exist because a certain number of methylated regions are not associated with siRNA expression in the Arabidopsis genome [9]. Small RNAs with homology to the 35S enhancer sequence could not be detected in either transgenic or wild-type gentians (Figure S6A) [11], therefore the *de novo* methylation of the 35S sequence might involve mechanisms other than the RdDM pathway. While no studies have identified an alternative coordinator in plants, possible mechanisms were proposed in mammals: that *de novo* methyltransferases themselves might recognize particular DNA or chromatin; and that *de novo* methyltransferases might be recruited through protein-protein interactions with transcriptional repressors or other factors [38]. More recently, research on the Arabidopsis *ibm1* mutation suggested that novel transcription-coupled mechanisms direct gene body methylation [39], hence such RdDM-independent target methylation mechanisms might exist in plants. Accordingly, we found nuclear factors that bind to the highly *de novo* methylated region (−149 to −124) of the 35S enhancer sequence in gentian. The form of the binding complex in gentian was distinct from that in tobacco (Figure 5B); therefore, some of the gentian nuclear factors might contribute to *de novo* methylation.

With regard to host genome defense [3], 35S methylation is thought to be a specially diversified system in gentian, contributing to genomic homeostasis against parasitic sequences. In the previous study, we showed that hypomethylation was found in the wild-type gentian genomic regions corresponding to those adjoining the T-DNA boundary of the transgenic plants [11], suggesting that the T-DNA was not entirely integrated into heterochromatin regions. In this regard, the 2C nuclear DNA content of gentian used in our experiment (10.57 pg) [40] is similar to that of tobacco (11.71 pg) [41]. No 35S-like sequences were detected in the wild-type gentian genome by Southern analysis (Figure S6B); therefore, it is much less likely that a virus sequence had been acquired in the ancestral gentian genome, comparable to the endogenous pararetroviruses in tobacco [42]. We thereby assume that *cis*-element(s) located within the *de novo* methylated region might be recognized as parasitic sequences. To confirm these possibilities, determination of the whole genome sequence of gentian would be required in the future.

### *De novo* methylation in the modified 35S promoters

Distinct distribution patterns of CpHpH methylation were observed in both petunia and gentian *CHS* core promoter regions, respectively. In the 35S(*GtCHS*) lines, slight CpHpH methylation was shown in the converted gentian *CHS* core promoter regions, which is consistent with the CpHpH hypomethylation of the intrinsic *CHS* promoter in wild-type gentian (Figure 4). Paradoxically, this result supports the existence of possible methylation machinery that acts sequence-specifically. On the other hand, the CpHpH methylation states of the petunia *CHS* core promoter regions varied among the 35S(*PhCHS*) lines (Figure 4). The variation of the CpHpH methylation patterns resembled those of the 35S(*nos-1*) lines, suggesting that their supplementary sequences involving *cis*-elements (i.e. *nos-1* and TACPyAT boxes) might affect *de novo* methylation directly or indirectly. Another possibility is that these additional sequences might occasionally permit spreading of *de novo* methylation activity by modification of histone tails. In any case, the variations of the distributions of CpHpH methylation in the modified 35S promoters make it difficult to reconcile the assumption that endogenous siRNAs with homology to the 35S enhancer region cause the *de novo* methylation. Even though further experiments are required to uncover the *de novo* methylation machinery, the present identification of the target region of *de novo* methylation impels us to search for the causal factor, which binds to the sequence and probably attracts a *de novo* methyltransferase.

## Materials and Methods

### Production of transgenic gentians

A binary vector, pSMABR35SsGFP, was constructed from pSMAB704 [43], by replacing the *uidA* coding sequence with the *sGFP* coding sequence [44] (Figure 1A). The T-DNA construct contains a CaMV-35S promoter-driven *sGFP* ORF with the NOS (nopaline synthase) terminator, and NOS promoter-driven *bar* (bialaphos resistance gene) [45] ORF with the Arabidopsis *rbcS* (rubisco small subunit) terminator. Modified 35S promoters were made from the pSMABR35SsGFP binary vector by replacing its *Xba*I/*Bam*HI/*Eco*RV sites within the 35Spro with each modified fragment (Figure 1A). Binary vectors were introduced into *Agrobacterium* strain EHA101 [46].

Transgenic gentian (*Gentiana triflora* × *G. scabra* cv. Polano-White) and tobacco (*Nicotiana tabacum* cv. SR1) plants were obtained by *Agrobacterium*-mediated transformation as described in our previous study [11]. Transgenic gentian and tobacco plants were maintained *in vitro* by subculturing the shoots (approx. 20 mm lengths) on 0.25% (w/v) gellan gum-solidified MS medium containing 3% (w/v) sucrose every three months and two months, respectively.

### Southern and northern blot analyses

For Southern blot analysis and bisulfite genomic sequencing, genomic DNAs were isolated from young leaves of *in vitro* growing plants using a GenElute Plant Genomic DNA Miniprep kit (Sigma-Aldrich) following the supplier's instructions. *Hin*dIII-digested genomic DNAs (5-µg aliquots) were separated by electrophoresis on 0.8% (w/v) agarose gels, blotted onto nylon membranes, and then fixed by UV irradiation. The blots were hybridized with *bar* and *sGFP* gene probes, which were prepared by the AlkPhos Direct Labeling System (GE Healthcare) following the supplier's instructions.

For northern blot analysis, total RNAs were isolated from young leaf tissues using the Concert Plant RNA reagent (Invitrogen) following the supplier's instructions. The isolated total RNAs (10 µg) were separated by electrophoresis on 1.2% (w/v) agarose-formaldehyde gels, blotted onto nylon membranes, and then fixed by UV irradiation. The same probes as used for Southern analysis were also used for northern hybridization. Hybridization, membrane washing and detection procedures were performed following the supplier's instructions (AlkPhos Direct Detection System; GE Healthcare).

To prepare *bar* and *sGFP* gene probes, the following primer pairs were used. For the 389-bp of *bar* gene ORF fragment, 5′-GGATCCATGAGCCCAGAACG-3′ (forward; F) and 5′-AGCCCGATGACAGCGACCAC-3′ (reverse; R) were used. For the 706-bp of *sGFP* gene ORF fragment, 5′-TGGTGAGCAAGGGCGAG-3′ (F) and 5′-TCGTCCATGCCGAGAGTGAT-3′ (R) were used.

### Bisulfite genomic sequencing

Bisulfite genomic sequencing [20] was performed as described in our previous study [11], or by using an EpiTect Bisulfite Kit (Qiagen) following the supplier's instructions, except for the bisulfite reaction conditions. For the EpiTect Bisulfite Kit, the bisulfite conversion reaction was performed in a thermal cycler as follows: 99.9°C for 10 min, 60°C for 25 min, 99.9°C for 10 min, 60°C for 85 min, 5 cycles of 99.9°C for 10 min and 60°C for 180 min, then maintaining at 20°C until the next step.

PCR reaction using bisulfite-treated genomic DNA as a template and subsequent cloning and sequencing was carried out as described in the previous study [11], except that the pSTBlue-1 vector (Novagen) was used for cloning. PCR was performed in 15 µl of AccuPrime Taq buffer II (Invitrogen) containing primers for top-strand amplification (concentrations of each primer are shown in Table S2), 0.5 µl AccuPrime Taq polymerase, and a 1–2 µl aliquot of bisulfite-treated DNA. The PCR reaction was performed as follows: 95°C for 2 min 30 sec, 5 cycles of 95°C for 30 sec, 55°C for 35 sec and 68°C for 1 min 30 sec, and 35 cycles of 95°C for 30 sec, 55°C for 35 sec and 68°C for 1 min.

### EMSA

Nuclear isolation was performed based on the methods of CelLytic™ PN (Sigma-Aldrich) and [28] with some modifications. Five grams of young leaves of wild-type gentian or tobacco plants were frozen in liquid nitrogen and ground to a powder. The powdered tissues were incubated with 40 ml of cold extraction buffer A [50 mM HEPES (pH 7.5), 0.4 M Sucrose, 5 mM MgCl_2_, 5 mM dithiothreitol (DTT), 0.1 mM phenylmethylsulfonyl fluoride (PMSF) and 0.4 mM Pefabloc SC] for 15 min on ice. The suspension was filtrated through two layers of Miracloth (Calbiochem), and centrifuged for 10 min at 700×g. The pellet was gently re-suspended in 1000 µl of extraction buffer B [extraction buffer A with 0.3% (v/v) Triton X-100], and the suspension was slowly transferred onto a 1500 µl of sucrose cushion (extraction buffer B with 1.8 M sucrose) and centrifuged for 10 min at 12000×g. The supernatant was removed, and the pellet was re-suspended in 60 µl of lysis buffer [20 mM HEPES (pH 7.9), 0.5 M NaCl, 20% (v/v) glycerol, 0.1 mM EDTA, 1mM DTT, 0.1 mM PMSF and 0.4 mM Pefabloc SC] and incubated for 30 min on ice. The suspension was centrifuged for 10 min at 12,000×g, and the supernatant was desalted using Micro Bio-Spin 6 Chromatography columns (Bio-Rad). All steps were carried out at 4°C.

Production of DIG 3′ end-labeled probes and detection of shift bands were performed by the DIG Gel Shift Kit, 2nd Generation (Roche). The oligonucleotides used for EMSA are listed in Table S3. Five micrograms of nuclear extracts were used for each reaction. Binding reactions (20 µl) contained 10 fmol of DIG 3′ end-labeled probe, 20 mM HEPES (pH 7.6), 1 mM EDTA, 10 mM (NH_4_)_2_SO_4_, 1 mM DTT, 0.2% (v/v) Tween 20, 50 mM KCl, 5 mM MgCl_2_, 2 µg poly(dI-dC) and competitor DNA. Five microliters of loading buffer [0.25×TBE, 60% (v/v) glycerol] was added to each reaction after 20 min of incubation at 25°C. Reactions were separated by electrophoresis on a 4% polyacrylamide gel in 0.5×TBE buffer at 4°C, electric blotted onto a nylon membrane (Biodyne PLUS; PALL), and fixed by UV irradiation.

## Supporting Information

Figure S1 GFP expression of the transgenic gentian calli. GFP fluorescence images of unmodified or modified 35Spro introduced transgenic gentian callus lines were obtained by FluorImager595 using 530DF30 filter with argon ion laser excitation (488nm). Untransformed gentian callus was used as a negative control (below center; bar = 10 mm). All the transgenic lines (line numbers are indicated at lower left) were single copy.
(0.46 MB PDF)

Figure S2 Representation of CpG, CpWpG and CpHpH methylation of the (modified) 35S-*sGFP* regions in the single copy transgenic gentians. (A–H) Cytosine methylation was analyzed in the unmodified 35S (A), 35S(Δ*as-1*) (B), 35S(*nos-1*) (C), 35S(*PhCHS*) (D), 35S(*GtCHS*) (E), 35S(Δcore) (F) and 35S core (G) transgenic gentian plants. Unmodified 35S transgenic tobacco plants were also analyzed as a control (H). Cytosine methylation patterns of the 35S enhancer region (−244 to −41) on the complementary (lower) strands were also analyzed in the unmodified 35S gentian lines #3, #5, #8 and #15 (I). The percentage of methylated cytosine is represented by bar charts (red, CpG; green, CpWpG; black, CpHpH), and each position of cytosines are represented below (black, 35Spro; blue, *sGFP*). Positions of start codons are indicated in pink, and positions of the known elements within the promoter regions are indicated by different colors (TATA box, aqua; *as-1*, olive; *as-2*, orange).
(1.15 MB PDF)

Figure S3 Expressions of the *sGFP* and *bar* transgenes in the modified 35S transgenic gentians. Northern blot analyses of *sGFP* and *bar* transgenes in leaf tissues of the single copy 35S(*nos-1*) (A), 35S(*PhCHS*) (B), 35S(*GtCHS*) (C), 35S core and 35S(Δcore) (D) transgenic gentian plants are shown. Wild-type (WT) gentian plant and unmodified 35S transgenic tobacco plant line #26 (designated as C) was used as a control. For comparison, unmodified 35S transgenic gentian plant #8 (35S-8) was also analyzed.
(3.13 MB PDF)

Figure S4 Representation of CpG, CpWpG and CpHpH methylation of NOS-*bar* and *rbcS*-35S regions in the unmodified 35S transgenic gentian lines, #15 and #19. (A) A schematic diagram showing NOS-*bar* (B) and *rbcS*-35S (C) regions for methylation analysis on T-DNA of the unmodified 35S-*sGFP* construct. (B, C) Cytosine methylation status of NOS promoter (black) with *bar* coding (blue) (B) and Arabidopsis *rbcS* terminator (green) with 35Spro (black) (C) regions. The percentage of methylated cytosines is represented by bar charts (red, CpG; green, CpWpG; black, CpHpH), and the position of each cytosine is represented below. Positions of the start codon and TATA-box are indicated in pink and aqua, respectively, and positions of the known elements within the promoter regions are indicated by different colors.
(0.13 MB PDF)

Figure S5 CpHpH methylation states of 35S(Δcore) lines in a time-series of culture. Percent methylation at CpHpH sites of the 35S(Δcore) lines, from which the genomic DNAs were obtained on Oct. 16, 2006 (black bars) and Mar. 22, 2007 (gray bars; the same data is represented on Figure 4), respectively, is shown. Analyzed regions of the 35S(Δcore) promoter with *sGFP* (black bar; indicated by arrows) are represented below. Positions of cytosines corresponding to the sequence from −148 to −85 of the unmodified 35S pro are indicated by red and pale red, respectively.
(0.11 MB PDF)

Figure S6 Small RNA and genomic DNA analyses to identify the endogenous 35S enhancer sequence in gentian. (A) Small-molecule RNAs from leaf tissues of wild-type (WT) and unmodified 35S-*sGFP* line #8 of gentian plants, and 35S-*sGFP* tobacco line #26 (C) were electrophoresed and hybridized with DIG-labeled 35S enhancer (upper) and 5S rRNA (lower) probes. Ethidium-stained gel bands serve as loading controls. (B) A survey of 35S enhancer sequence in the gentian genome. Southern blot analysis was performed using *Hin*dIII-digested genomic DNAs of wild-type (WT) and unmodified 35S-*sGFP* (#8 of gentian and #26 of tobacco) transgenic gentian and tobacco plants hybridized with the 35S enhancer probe. Hybridization was performed with high (42°C; left panel) and low (37°C; right) stringency conditions.
(3.74 MB PDF)

Table S1 Summary of transgenic gentian production and Southern analysis.
(0.02 MB PDF)

Table S2 Primers used for bisulfite-PCR in the analysis of methylation of transgenes and endogenous genes in gentian.
(0.03 MB PDF)

Table S3 List of oligonucleotides used as EMSA probes and/or competitors.
(0.02 MB PDF)
