# Supplementary figures and images for: Strict De Novo Methylation of the 35S Enhancer Sequence in Gentian
Source: PLoS One. 2010 Mar 23;5(3):e9670. doi: 10.1371/journal.pone.0009670 (PMC2843634; doi:10.1371/journal.pone.0009670)

**Unmodified 35S**

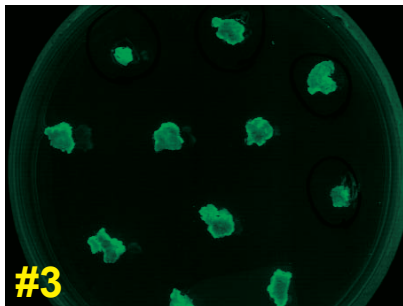

**35S(*nos-1*)**

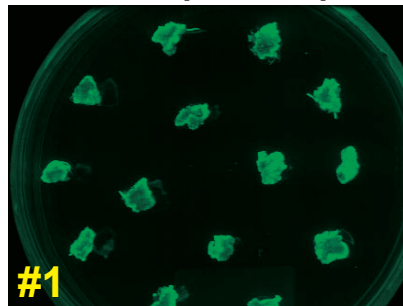

**35S(*PhCHS*)**

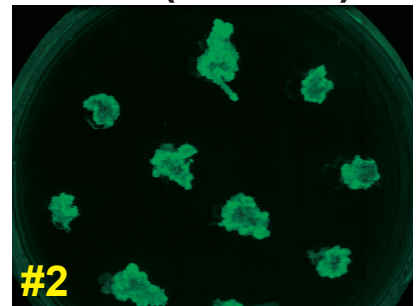

**35S core**

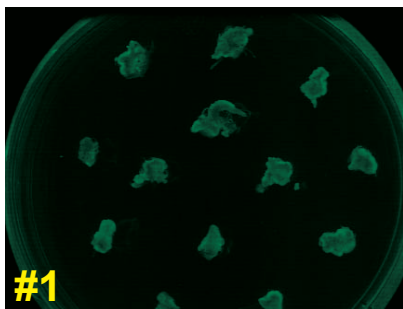

**35S( $\Delta$ *as-1*)**

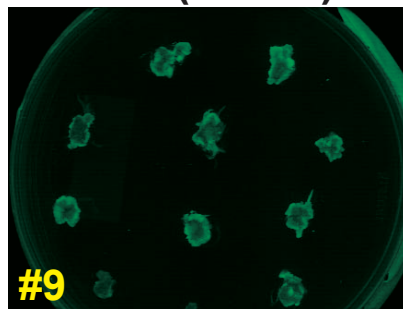

**35S(*GtCHS*)**

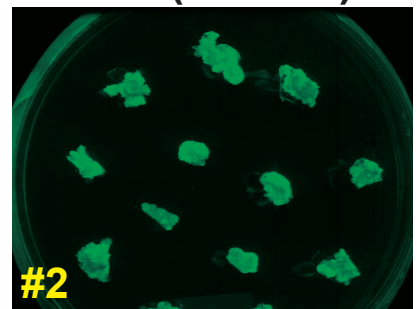

**Untransformed**

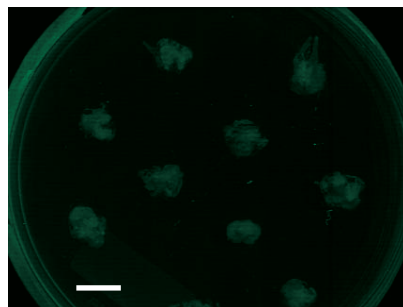

Supplement: Figure S1 — GFP expression of the transgenic gentian calli. GFP fluorescence images of unmodified or modified 35Spro introduced transgenic gentian callus lines were obtained by FluorImager595 using 530DF30 filter with argon ion laser excitation (488nm). Untransformed gentian callus was used as a negative control (below center; bar = 10 mm). All the transgenic lines (line numbers are indicated at lower left) were single copy. (0.46 MB PDF) [file pone.0009670.s001.pdf]

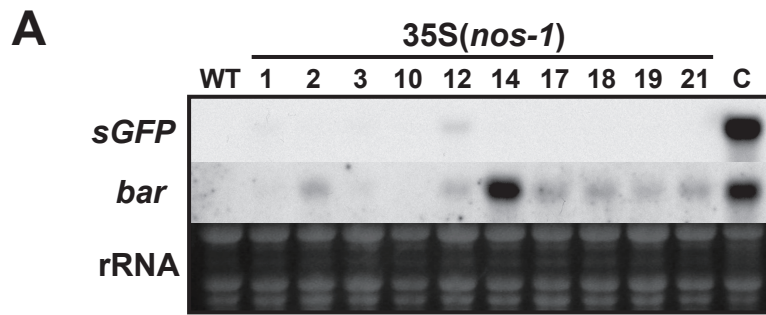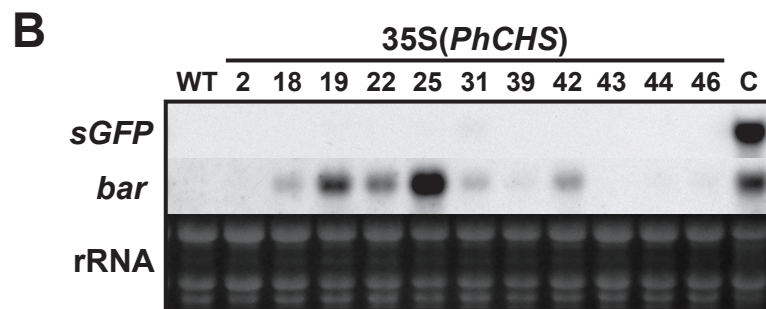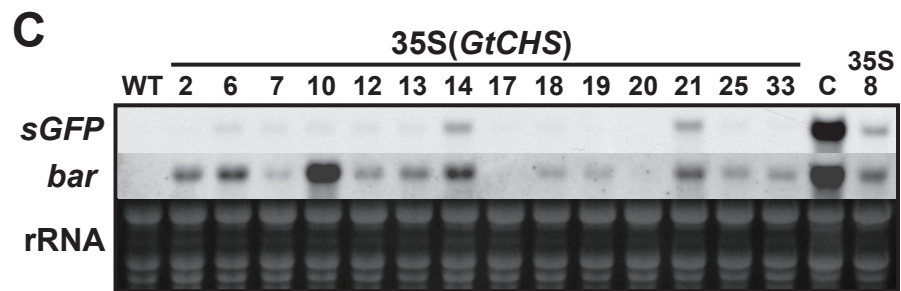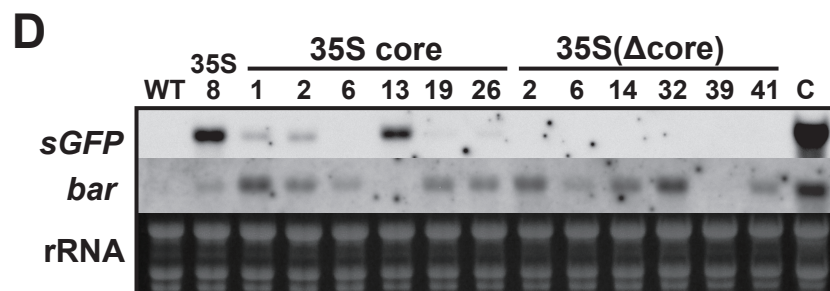

Supplement: Figure S3 — Expressions of the sGFP and bar transgenes in the modified 35S transgenic gentians. Northern blot analyses of sGFP and bar transgenes in leaf tissues of the single copy 35S(nos-1) (A), 35S(PhCHS) (B), 35S(GtCHS) (C), 35S core and 35S(Δcore) (D) transgenic gentian plants are shown. Wild-type (WT) gentian plant and unmodified 35S transgenic tobacco plant line #26 (designated as C) was used as a control. For comparison, unmodified 35S transgenic gentian plant #8 (35S-8) was also analyzed. (3.13 MB PDF) [file pone.0009670.s003.pdf]

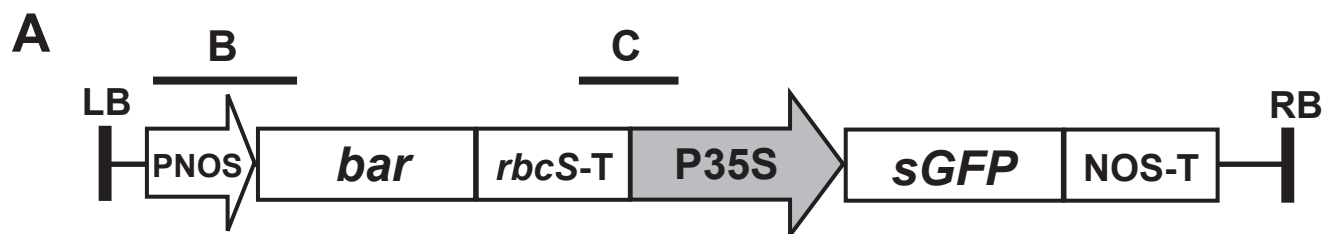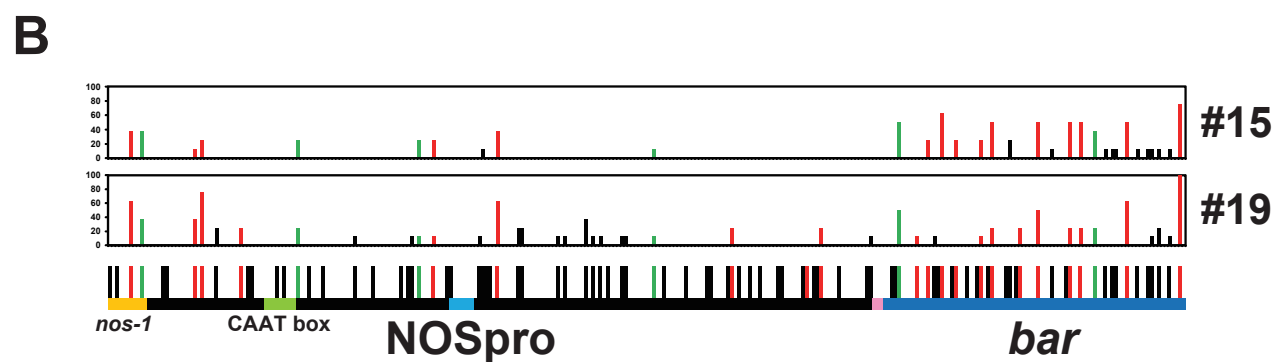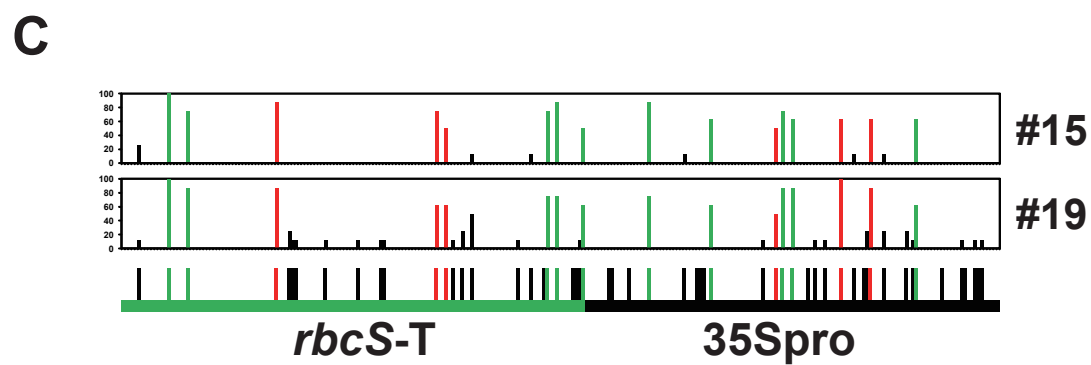

Supplement: Figure S4 — Representation of CpG, CpWpG and CpHpH methylation of NOS-bar and rbcS-35S regions in the unmodified 35S transgenic gentian lines, #15 and #19. (A) A schematic diagram showing NOS-bar (B) and rbcS-35S (C) regions for methylation analysis on T-DNA of the unmodified 35S-sGFP construct. (B, C) Cytosine methylation status of NOS promoter (black) with bar coding (blue) (B) and Arabidopsis rbcS terminator (green) with 35Spro (black) (C) regions. The percentage of methylated cytosines is represented by bar charts (red, CpG; green, CpWpG; black, CpHpH), and the position of each cytosine is represented below. Positions of the start codon and TATA-box are indicated in pink and aqua, respectively, and positions of the known elements within the promoter regions are indicated by different colors. (0.13 MB PDF) [file pone.0009670.s004.pdf]

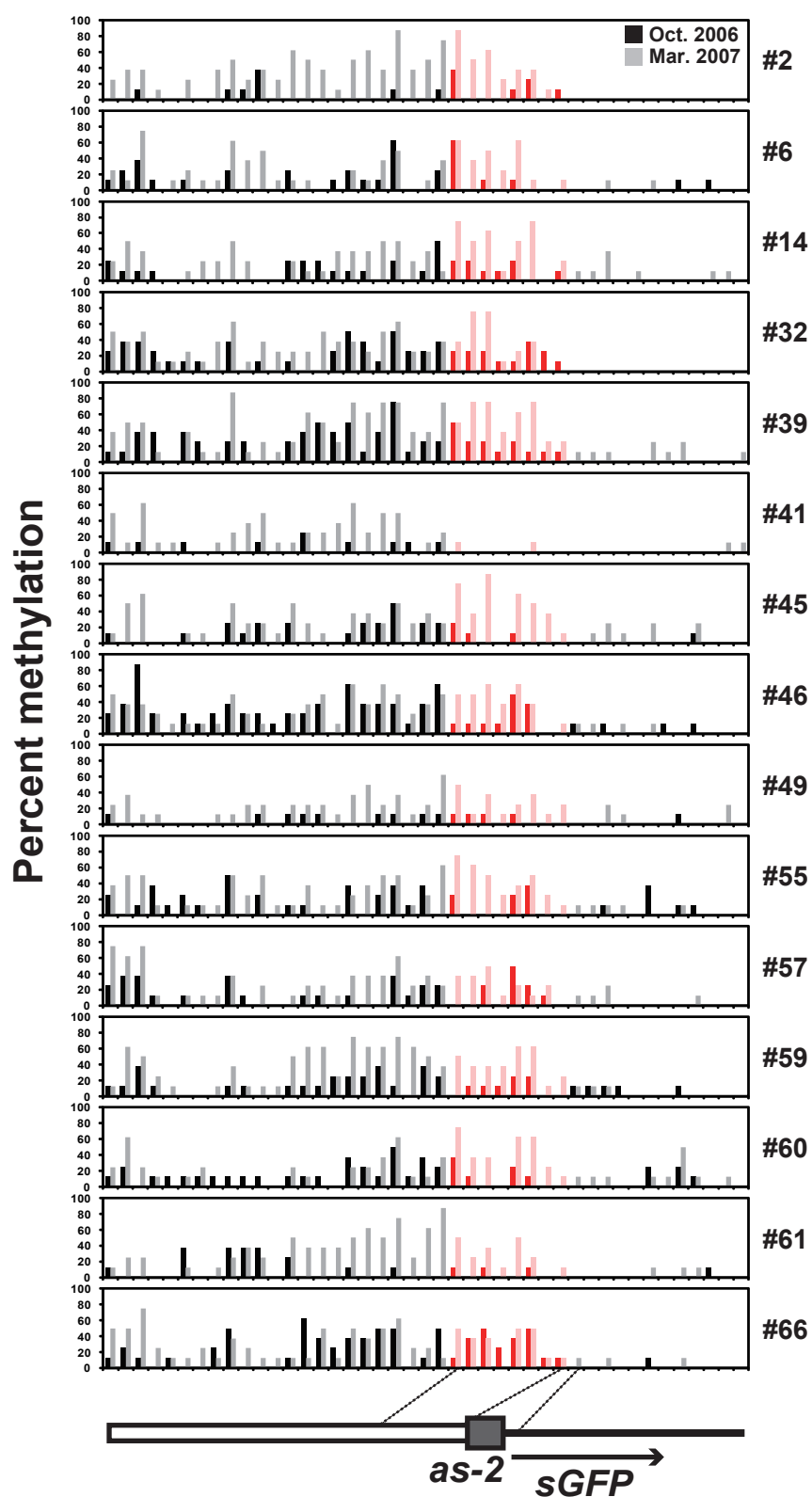

Supplement: Figure S5 — CpHpH methylation states of 35S(Δcore) lines in a time-series of culture. Percent methylation at CpHpH sites of the 35S(Δcore) lines, from which the genomic DNAs were obtained on Oct. 16, 2006 (black bars) and Mar. 22, 2007 (gray bars; the same data is represented on Figure 4), respectively, is shown. Analyzed regions of the 35S(Δcore) promoter with sGFP (black bar; indicated by arrows) are represented below. Positions of cytosines corresponding to the sequence from −148 to −85 of the unmodified 35S pro are indicated by red and pale red, respectively. (0.11 MB PDF) [file pone.0009670.s005.pdf]
