# Supplementary material for: Strict De Novo Methylation of the 35S Enhancer Sequence in Gentian
Source: PLoS One. 2010 Mar 23;5(3):e9670. doi: 10.1371/journal.pone.0009670 (PMC2843634; doi:10.1371/journal.pone.0009670)

A unmodified 35S

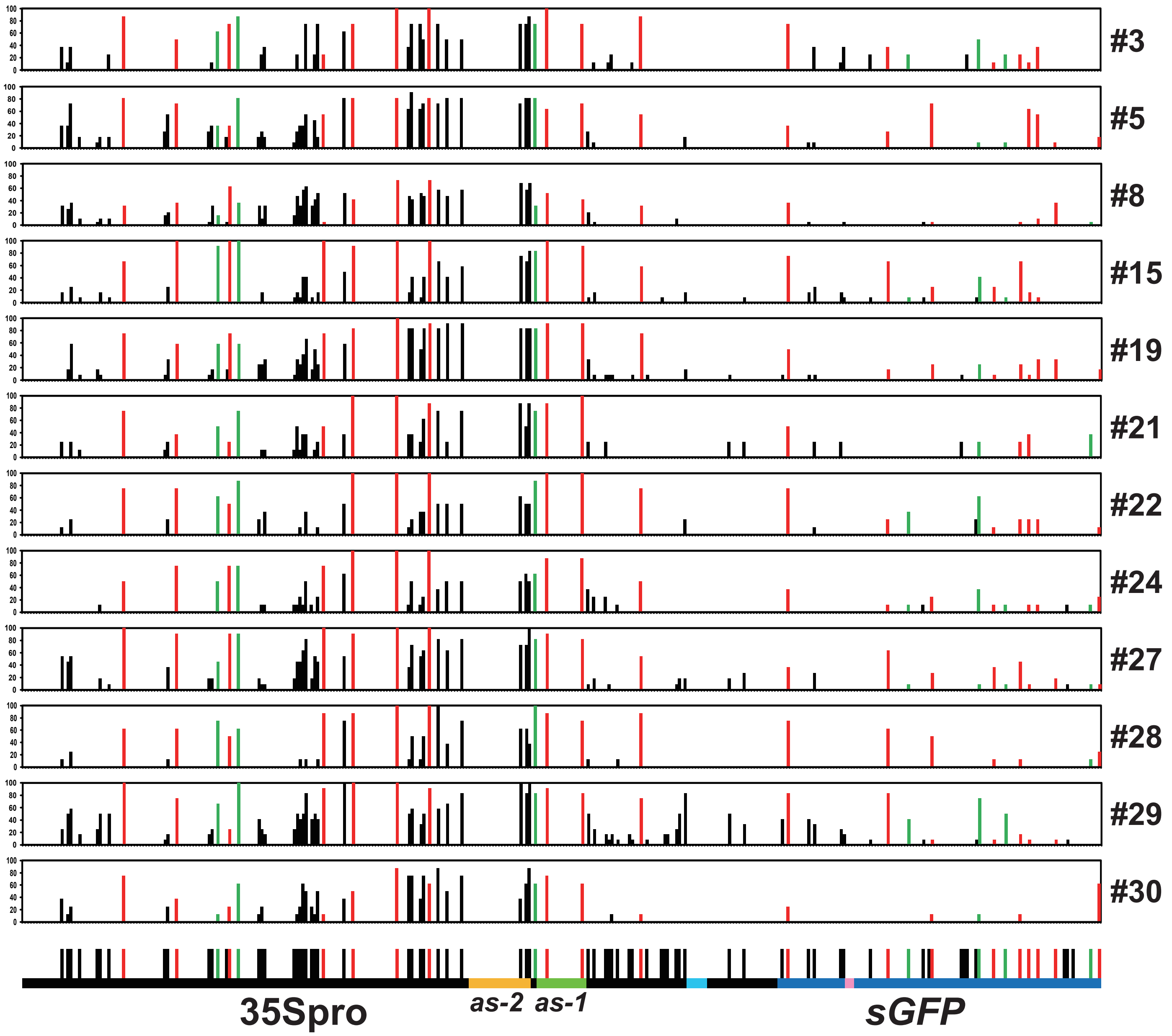

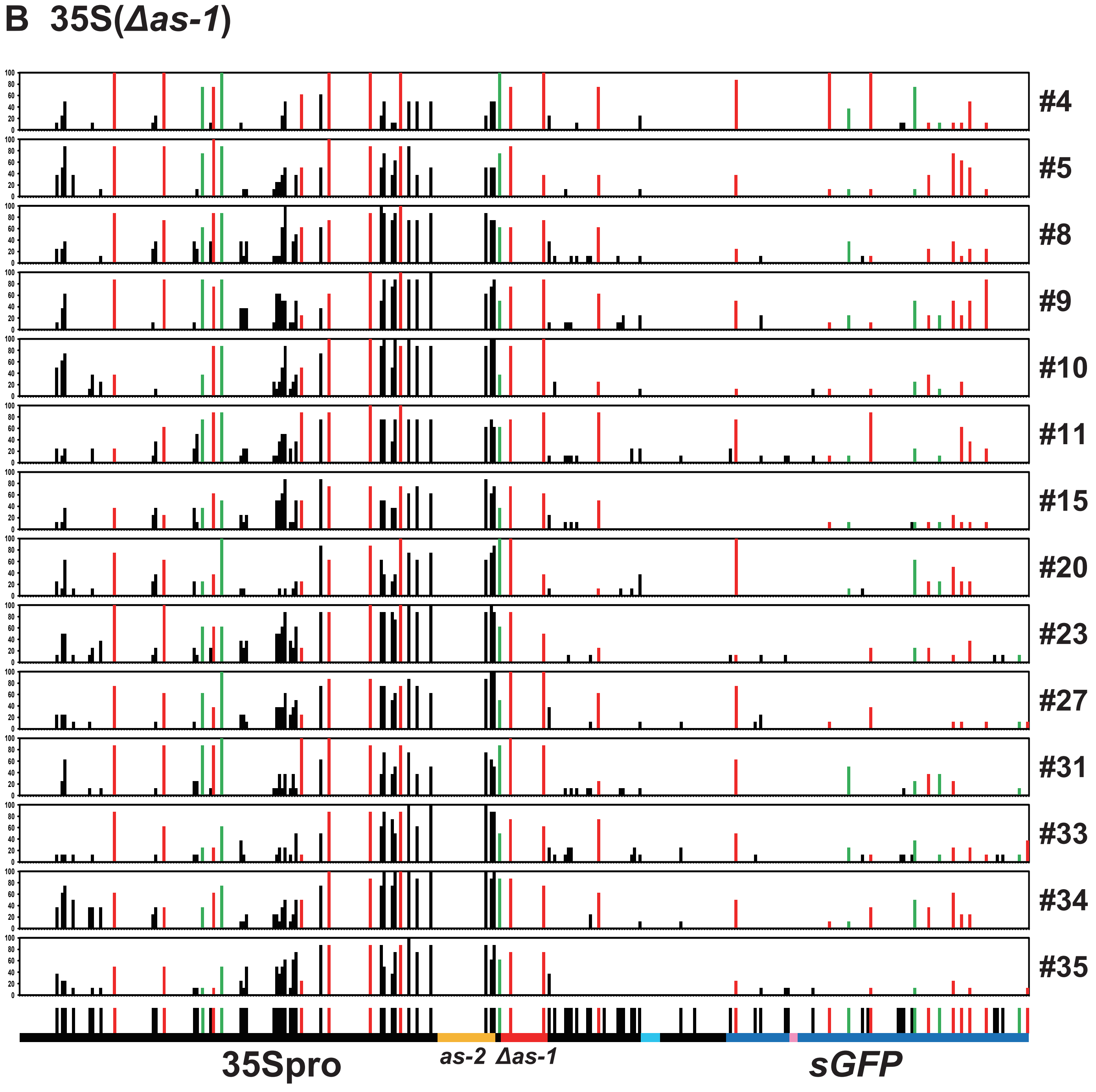
B 35S(Δas-1)
#4
#5
#8
#9
#10
#11
#15
#20
#23
#27
#31
#33
#34
#35
35Spro
as-2 Δas-1
sGFP

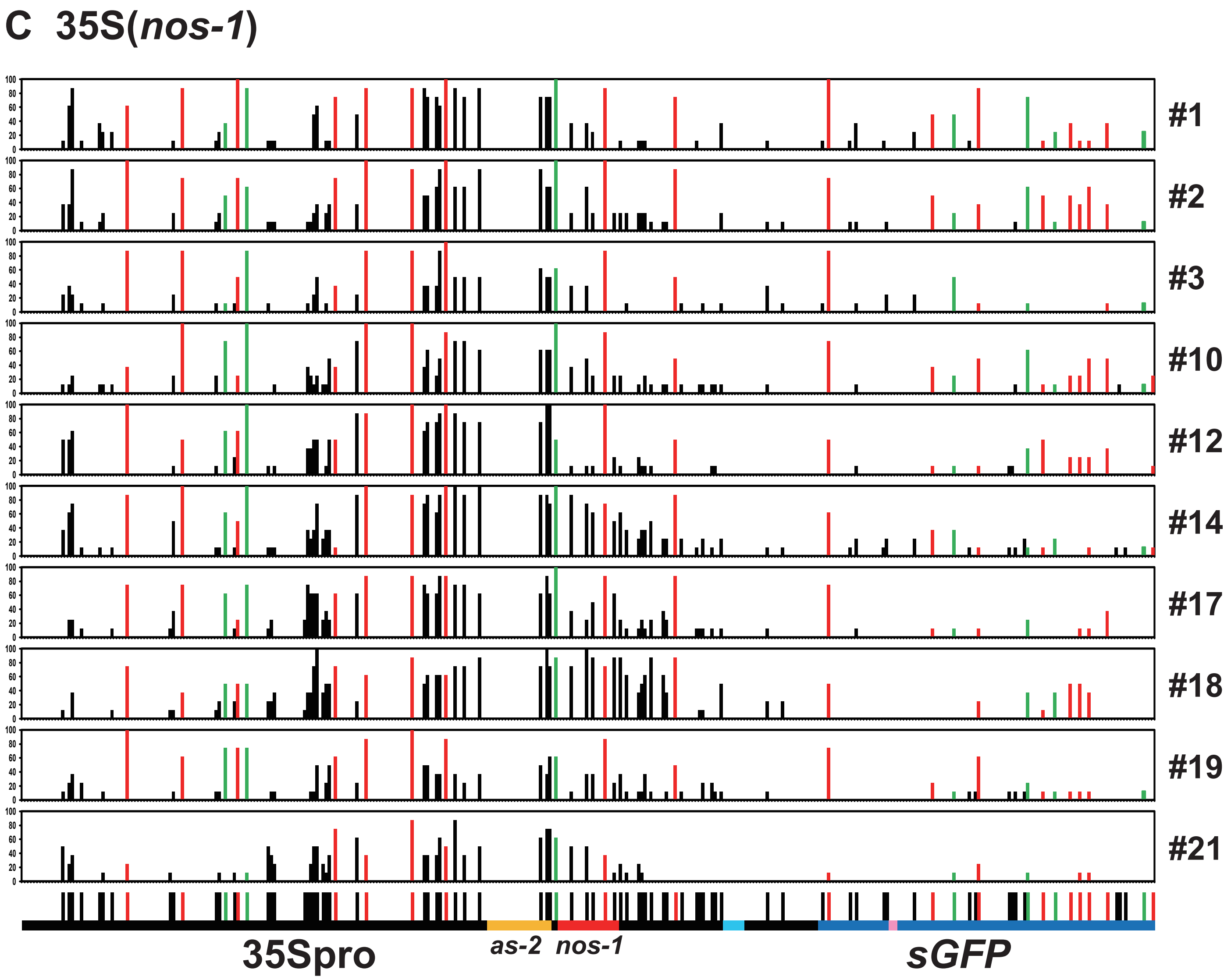

C 35S(nos-1)
#1
#2
#3
#10
#12
#14
#17
#18
#19
#21
35Spro
as-2
nos-1
sGFP

D 35S(*PhCHS*)

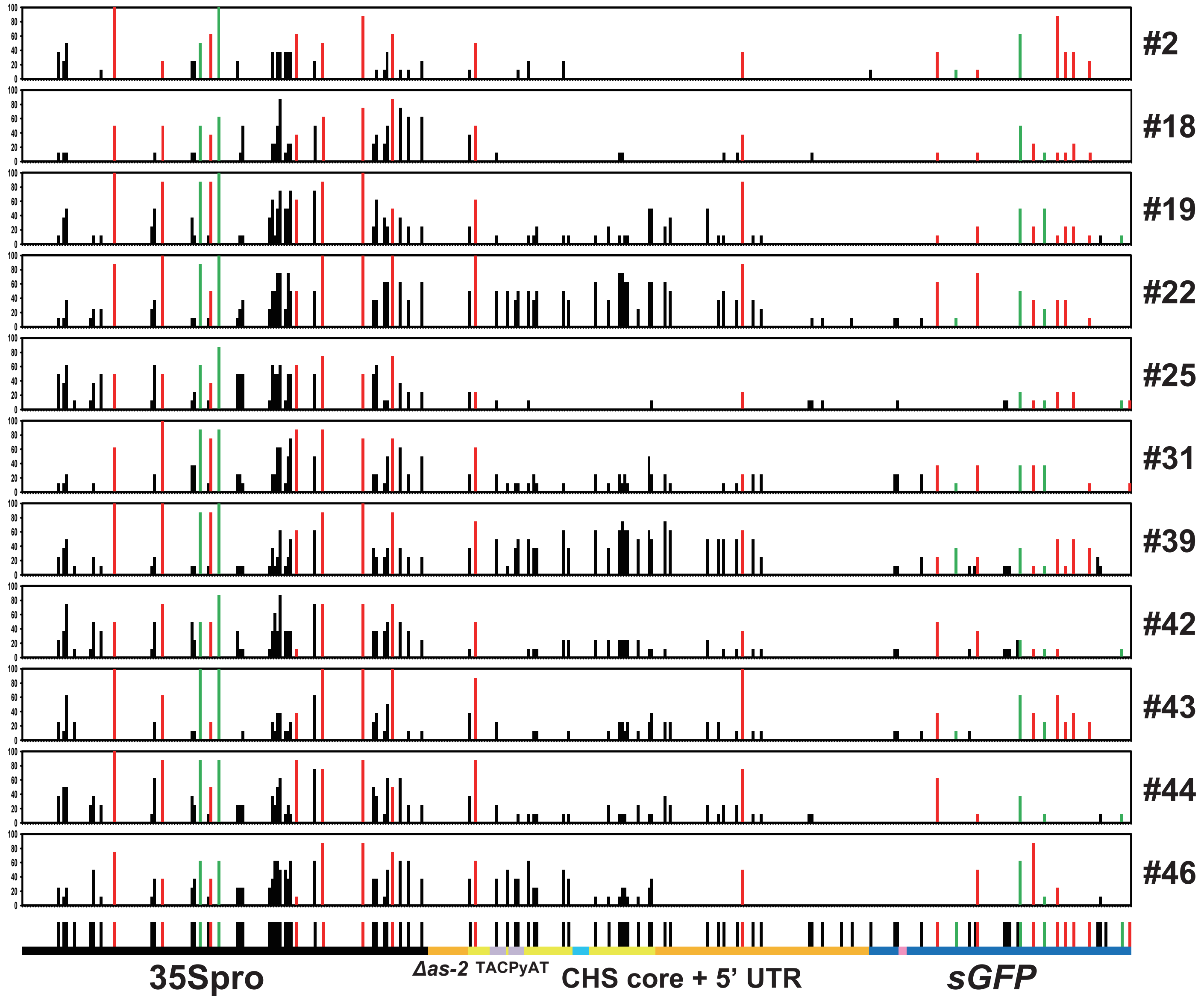

#2
#18
#19
#22
#25
#31
#39
#42
#43
#44
#46
35Spro
Δas-2 TACPyAT
CHS core + 5' UTR
sGFP

E 35S(*GtCHS*)

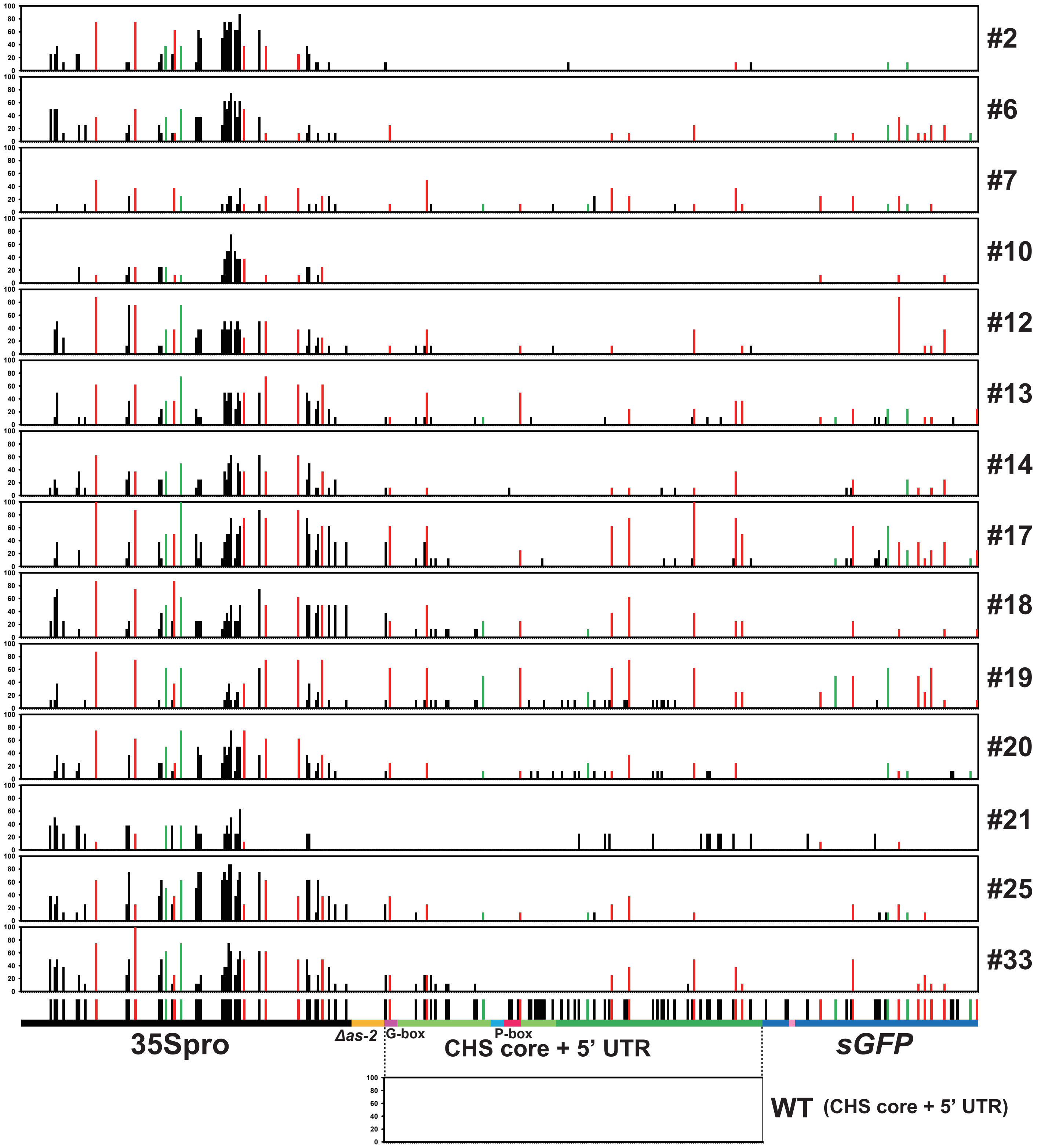

#2
#6
#7
#10
#12
#13
#14
#17
#18
#19
#20
#21
#25
#33
35Spro
Δas-2
G-box
P-box
CHS core + 5' UTR
sGFP
WT (CHS core + 5' UTR)

F 35S(Δcore)

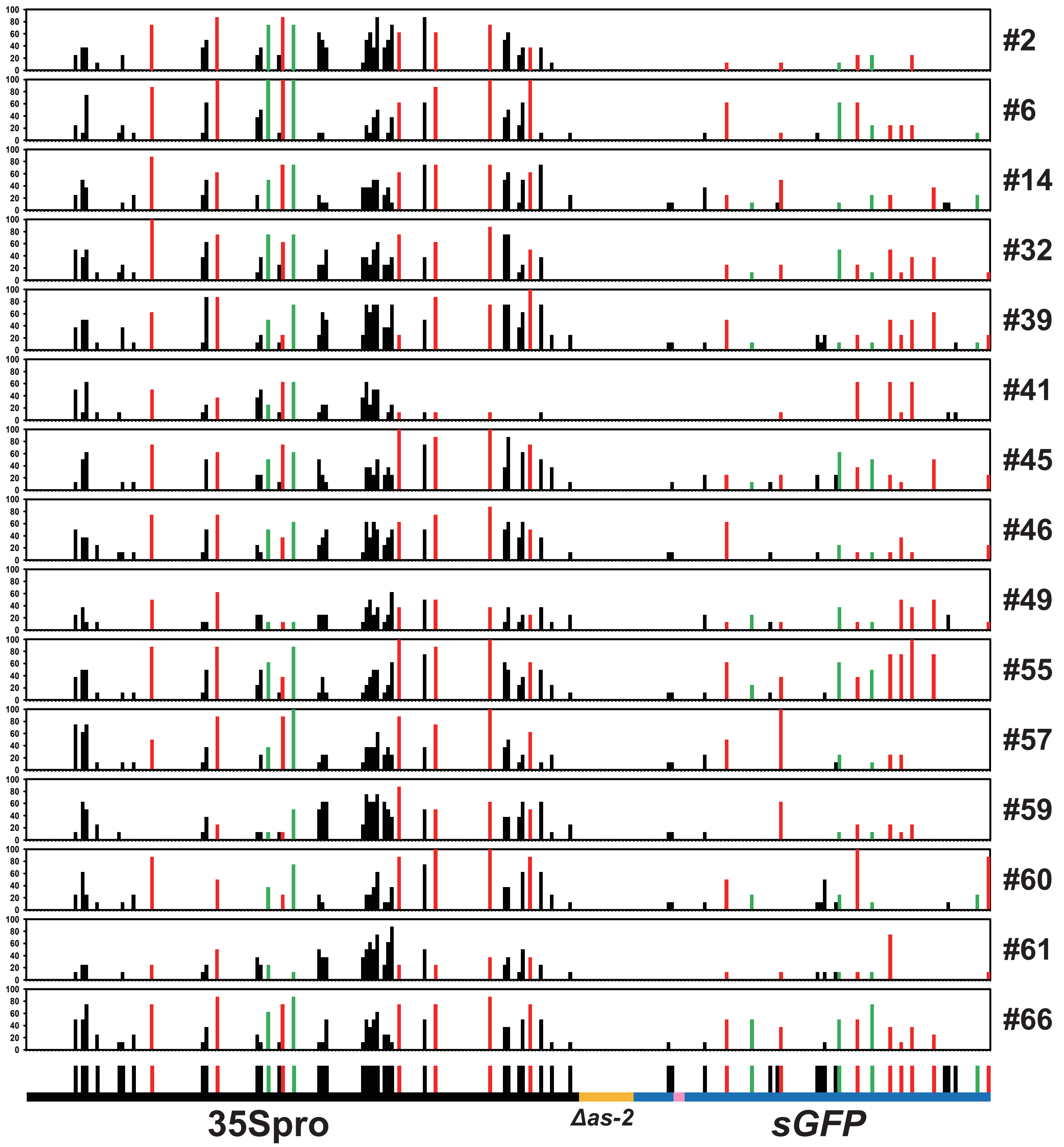
100
80
60
40
20
0
#2
#6
#14
#32
#39
#41
#45
#46
#49
#55
#57
#59
#60
#61
#66
35Spro
Δas-2
sGFP

G 35S core

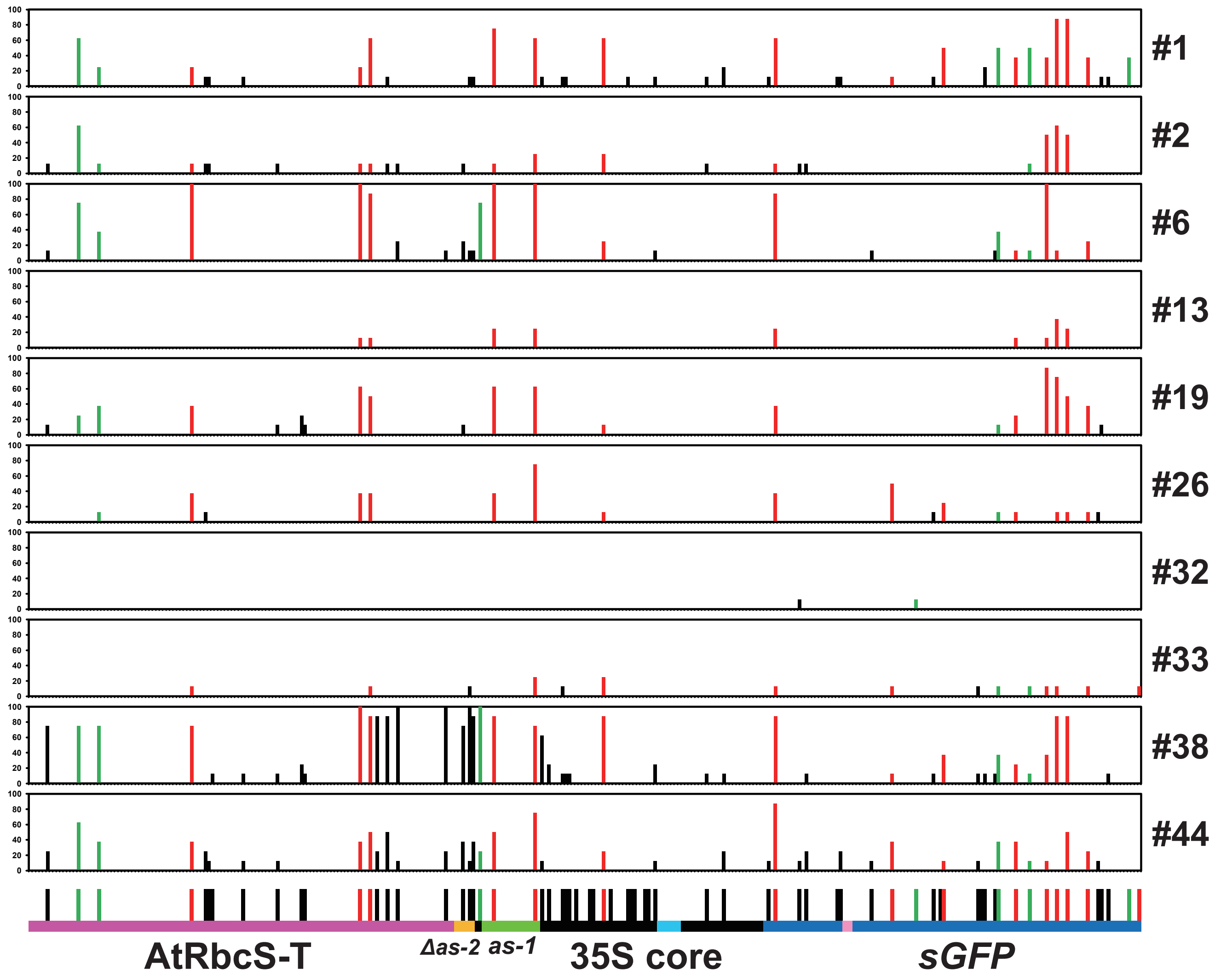

100
80
60
40
20
0
#1
#2
#6
#13
#19
#26
#32
#33
#38
#44
AtRbcS-T
Δas-2 as-1
35S core
sGFP

H unmodified 35S (transgenic tobacco)

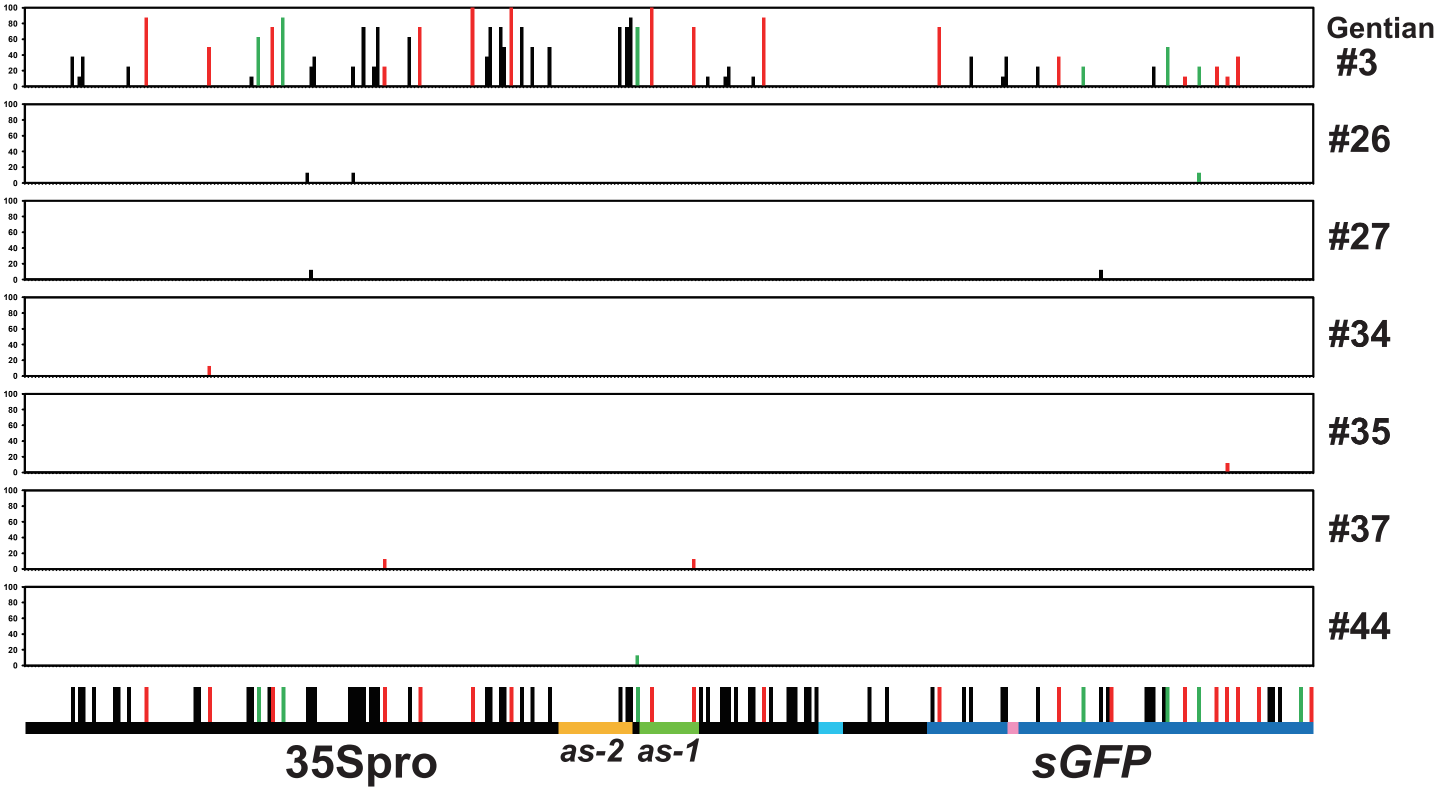

I unmodified 35S (with complementary strand)

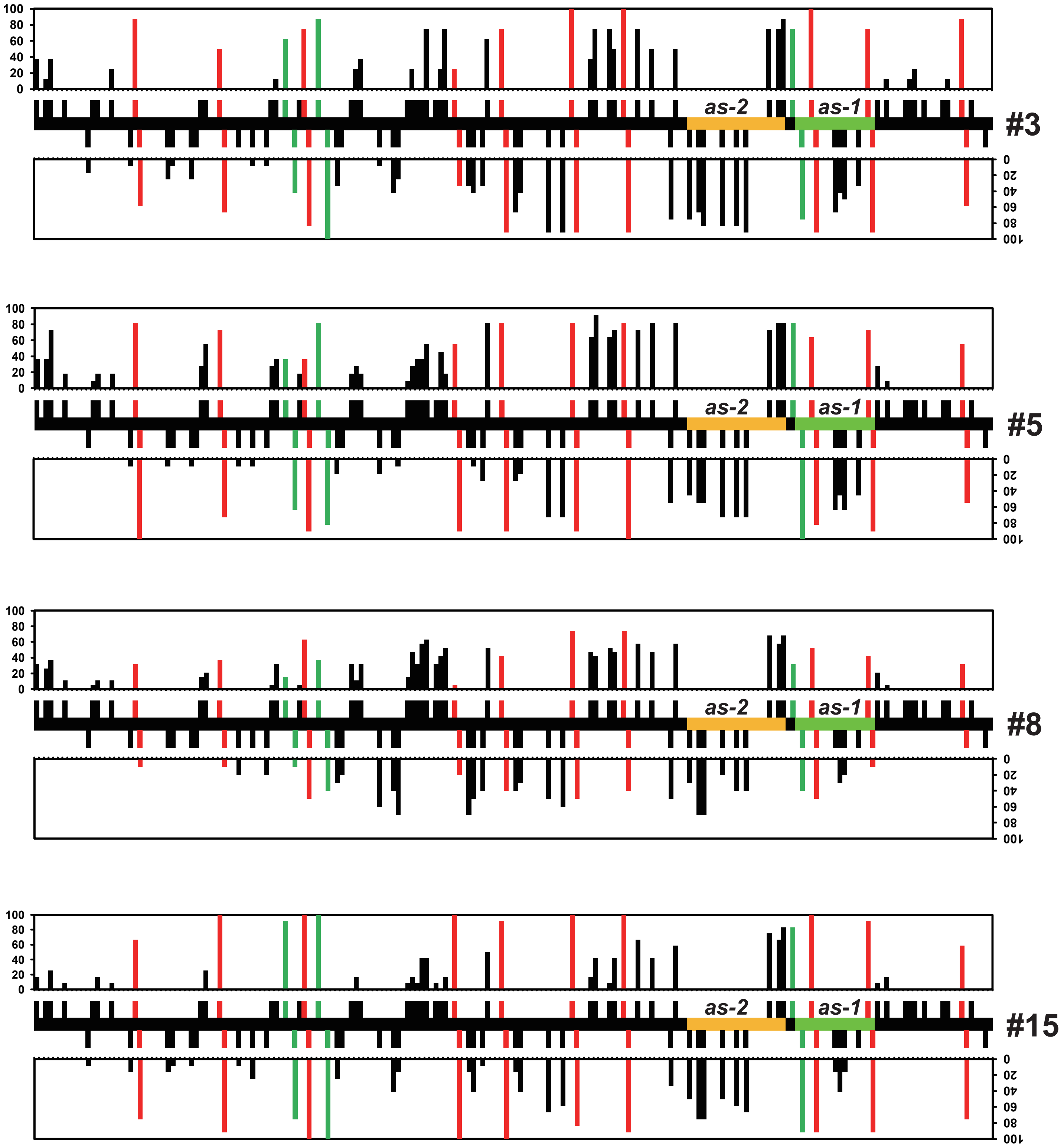

Supplement: Figure S2 — Representation of CpG, CpWpG and CpHpH methylation of the (modified) 35S-sGFP regions in the single copy transgenic gentians. (A–H) Cytosine methylation was analyzed in the unmodified 35S (A), 35S(Δas-1) (B), 35S(nos-1) (C), 35S(PhCHS) (D), 35S(GtCHS) (E), 35S(Δcore) (F) and 35S core (G) transgenic gentian plants. Unmodified 35S transgenic tobacco plants were also analyzed as a control (H). Cytosine methylation patterns of the 35S enhancer region (−244 to −41) on the complementary (lower) strands were also analyzed in the unmodified 35S gentian lines #3, #5, #8 and #15 (I). The percentage of methylated cytosine is represented by bar charts (red, CpG; green, CpWpG; black, CpHpH), and each position of cytosines are represented below (black, 35Spro; blue, sGFP). Positions of start codons are indicated in pink, and positions of the known elements within the promoter regions are indicated by different colors (TATA box, aqua; as-1, olive; as-2, orange). (1.15 MB PDF) [file pone.0009670.s002.pdf]
