# Supplementary material for: Strict De Novo Methylation of the 35S Enhancer Sequence in Gentian
Source: PLoS One. 2010 Mar 23;5(3):e9670. doi: 10.1371/journal.pone.0009670 (PMC2843634; doi:10.1371/journal.pone.0009670)
Supplement: Figure S6 — Small RNA and genomic DNA analyses to identify the endogenous 35S enhancer sequence in gentian. (A) Small-molecule RNAs from leaf tissues of wild-type (WT) and unmodified 35S-sGFP line #8 of gentian plants, and 35S-sGFP tobacco line #26 (C) were electrophoresed and hybridized with DIG-labeled 35S enhancer (upper) and 5S rRNA (lower) probes. Ethidium-stained gel bands serve as loading controls. (B) A survey of 35S enhancer sequence in the gentian genome. Southern blot analysis was performed using HindIII-digested genomic DNAs of wild-type (WT) and unmodified 35S-sGFP (#8 of gentian and #26 of tobacco) transgenic gentian and tobacco plants hybridized with the 35S enhancer probe. Hybridization was performed with high (42°C; left panel) and low (37°C; right) stringency conditions. (3.74 MB PDF) [file pone.0009670.s006.pdf]

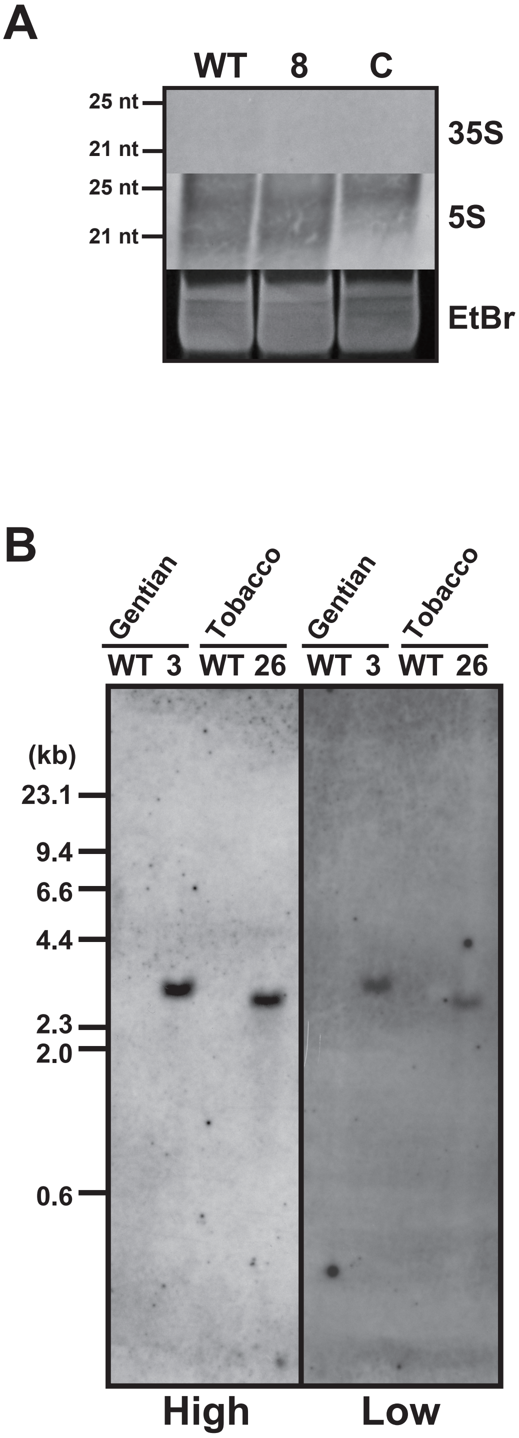

A
WT
8
C
25 nt
21 nt
35S
25 nt
21 nt
5S
EtBr
B
Gentian
Tobacco
Gentian
Tobacco
WT 3 WT 26 WT 3 WT 26
(kb)
23.1
9.4
6.6
4.4
2.3
2.0
0.6
High
Low

Experimental procedures for supplementary data

Small RNA and genomic DNA analyses for 35S enhancer sequence detection

Low molecular weight RNAs were isolated from each 0.4 g F.W. of young leaf tissues of wild-type and 35S-*sGFP* gentians and tobacco plants using a High Pure miRNA Isolation Kit (Roche). The low molecular-weight RNA samples were separated by 15% PAGE at 180V, and the gel was transferred to a Biodyne A (PALL) nylon membrane with a semidry blotter. After transfer, 1-ethyl-3-(3-dimethylaminopropyl) carbodiimide (EDC)-mediated cross-linking (Pall and Hamilton, 2008) was performed for 2h at 60 ºC. Hybridization and non-radioactive detection of small RNAs were performed as described by Goto et al. (2003). DIG-labeled 35S enhancer (278-bp; -355 to -78) and 5S rRNA gene (114-bp) probes were prepared by PCR using primers as follows: for 35S enhancer, 5'-CCAAAGGGCAATTGAGACTTTT-3' and 5'-CGTCAGTGGAGATATCACATCAA-3'; for 5S, 5'-TGCGATCATACCAGCACTAA-3' and 5'-GRTGCAACACRAGGACTTCCC-3'.

For Southern analysis, genomic DNAs of gentian and tobacco plants were isolated as described above and each 5 μg was used. Hybridization, membrane washing and detection procedures were performed using DIG-labeling system (Roche) following the supplier’s instructions. Different hybridization conditions (at 42 ºC and 37 ºC) were tested using the DIG-labeled 35S enhancer probe as described above.

References

Goto K, Kanazawa A, Kusaba M, Masuta C (2003) A simple and rapid method to detect plant siRNAs using nonradioactive probes. Plant Mol Biol Rep 21: 51-58.

Pall GS, Hamilton AJ (2008) Improved northern blot method for enhanced detection of small RNA. Nat Protocol 3: 1077-1084.
