# Supplementary material for: Strict De Novo Methylation of the 35S Enhancer Sequence in Gentian
Source: PLoS One. 2010 Mar 23;5(3):e9670. doi: 10.1371/journal.pone.0009670 (PMC2843634; doi:10.1371/journal.pone.0009670)
Supplement: Table S1 — Summary of transgenic gentian production and Southern analysis. (0.02 MB PDF) [file pone.0009670.s007.pdf]

Table S1. Summary of transgenic gentian production and Southern analysis.

vectors	No. of transgenic plant lines	No. of lines having single band		No. of lines analyzed for DNA methylation
		bar	*sGFP*	
unmodified 35S	21	12	12	12
(Transgenic tobacco)	31	8	6	6
35S(Δ*as-1*)	29	14	16	14
35S(*nos-1*)	15	11	10	10
35S(*PhCHS*)	41	13	16	11
35S(*GtCHS*)	27	17	17	14
35S core	31	12	13	10
35S(Δcore)	51	16	22	15
