# Supplementary material for: Strict De Novo Methylation of the 35S Enhancer Sequence in Gentian
Source: PLoS One. 2010 Mar 23;5(3):e9670. doi: 10.1371/journal.pone.0009670 (PMC2843634; doi:10.1371/journal.pone.0009670)
Supplement: Table S2 — Primers used for bisulfite-PCR in the analysis of methylation of transgenes and endogenous genes in gentian. (0.03 MB PDF) [file pone.0009670.s008.pdf]

Table S2. Primers used for bisulfite-PCR in the analysis of methylation of transgenes and endogenous genes in gentian.

Target[a]	Origin[a]	Direction	Length	Sequence (5' to 3')[b]	Conc.[c]	Product[d]	Related figure(s)
35S-*sGFP*	transgene	forward	32	AGYTATYTGTYAYTTTATTGTGAAGATAGTGG	2	423[e]	Figs. 2, 3, 4, S2
		reverse	24	ATCRCCCTCRCCCTCRCCRRACAC	4		
rbcsT-*sGFP*	transgene	forward	34	TGGAAATTTTGTTTAYGTTAAATTTGATYATTGG	1	383	Figs. 4, S2 (35S core)
		reverse	24	ATCRCCCTCRCCCTCRCCRRACAC	4		
rbcsT-35S	transgene	forward	34	TGGAAATTTTGTTTAYGTTAAATTTGATYATTGG	1	332	Fig. S4
		reverse	29	TCCTRAATCTTTTRACTRCATCTTTAACC	2		
NOS-*bar*	transgene	forward	26	GGGTTTYTGGAGTTTAATGAGYTAAG	1	358	Fig. S4
		reverse	30	CTCRRTACRRAARTTRACCRTRCTTRTCTC	5		
35S complementary	transgene	forward	38	ACACACTTRTCTACTCCAAAAATATCAAARATACARTC	2	394	Fig. S2
		reverse	33	AAATGAAATGAAYTTYYTTATATAGAGGAAGGG	2		
endo-*chs*	endogenous	forward	25	ATTTGTTGGYTAGAYATGAGYTAGG	4	421	not shown
		reverse	27	TTCTRATCTCCTCAACRRTCACCATTC	2		

[a]LB, genome; T-DNA left border boundary region in gentian genome

[b]Y=C, T; R=A, G

[c]Primer concentration (μM) used for the reaction

[d]Amplified product length (bp)

[e]423 bp for unmodified 35S and 35S(*as-1*), 426 bp for 35S(nos-1), 471 bp for 35S(*PhCHS*), 496 bp for 35S(*GtCHS*), 300 bp for 35S(Δcore)
