# Supplementary material for: Strict De Novo Methylation of the 35S Enhancer Sequence in Gentian
Source: PLoS One. 2010 Mar 23;5(3):e9670. doi: 10.1371/journal.pone.0009670 (PMC2843634; doi:10.1371/journal.pone.0009670)
Supplement: Table S3 — List of oligonucleotides used as EMSA probes and/or competitors. (0.02 MB PDF) [file pone.0009670.s009.pdf]

Table S3. List of oligonucleotides used as EMSA probes and/or competitors.

Target[a]	Direction	Length	Sequence (5' to 3')[b]
-254 to -229	forward	26	AGGAAGGTGGCTCCTACAAATGCCAT
	reverse	26	ATGGCATTTGTAGGAGCCACCTTCCT
-233 to -208	forward	26	GCCATCATTGCGATAAAGGAAAGGCC
	reverse	26	GGCCTTTCCTTTATCGCAATGATGGC
-212 to -187	forward	26	AGGCCATCGTTGAAGATGCCTCTGCC
	reverse	26	GGCAGAGGCATCTTCAACGATGGCCT
-191 to -166	forward	26	CTGCCGACAGTGGTCCCAAAGATGGA
	reverse	26	TCCATCTTTGGGACCACTGTCGGCAG
-170 to -145	forward	26	ATGGACCCCCACCCACGAGGAGCATC
	reverse	26	GATGCTCCTCGTGGGTGGGGGTCCAT
-149 to -124	forward	26	GCATCGTGGAAAAAGAAGACGTTCCA
	reverse	26	TGGAACGTCTTCTTTTTCCACGATGC
-149 to -124 (mt)	forward	26	GCATCGTGGAAAAAGAAGA**TT**TTCCA
	reverse	26	TGGAA**AA**TCTTCTTTTTCCACGATGC
-128 to -103	forward	26	TTCCAACCACGTCTTCAAAGCAAGTG
	reverse	26	CACTTGCTTTGAAGACGTGGTTGGAA
-107 to -85 (as-2)	forward	25	AAGTGGATTGATGTGATATCTCCAC
	reverse	25	GTGGAGATATCACATCAATCCACTT

[a]Target regions indicated by positions in the 35S promoter

[b]Red letters indicate mutated nucleotides
